# Supplementary material for: Monocytic TLR4 expression and activation in schizophrenia: A systematic review and meta-analysis
Source: PLoS One. 2025 Mar 28;20(3):e0319171. doi: 10.1371/journal.pone.0319171 (PMC11952227; doi:10.1371/journal.pone.0319171)
Supplement: Supporting Information 2 — (PDF) [file pone.0319171.s002.pdf]

Supplementary Materials

Monocytic TLR4 expression and activation in schizophrenia: a systematic review and meta-analysis

**Table of Contents**		**Page**
**Table S1**	**The PRISMA2020 statement: An updated guideline for reporting systematic reviews**	**1-2**
**Table S2**	**The PRISMA 2020 for Abstracts Checklist**	**3**
**Table S3**	**Systematic search syntax for MEDLINE (via PubMed) with results**	**4**
**Table S4**	**Systematic search syntax for Web of Science with results**	**5**
**Table S5**	**Systematic search syntax for Scopus with results**	**6**
**Table S6**	**Studies identified in the literature search that proceeded to full-text screening**	**7-12**
**Table S7**	**Risk of bias evaluation, the Joanna Briggs Institute (JBI) Critical Appraisal tool**	**13**
**Figure S1**	**Galbraith plots for analysis of heterogeneity across studies**	**14**
**Figure S2**	**Funnel Plots for analysis of publication bias**	**15**
**References**		

Table S1. The PRISMA2020 statement: An updated guideline for reporting systematic reviews.

Section and Topic	Item #	Checklist item	Page
**TITLE**			
Title	1	Identify the report as a systematic review.	1
**ABSTRACT**			
Abstract	2	See the PRISMA 2020 for Abstracts checklist.	Table S2
**INTRODUCTION**			
Rationale	3	Describe the rationale for the review in the context of existing knowledge.	2-3
Objectives	4	Provide an explicit statement of the objective(s) or question(s) the review addresses.	3
**METHODS**			
Eligibility criteria	5	Specify the inclusion and exclusion criteria for the review and how studies were grouped for the syntheses.	5
Information sources	6	Specify all databases, registers, websites, organisations, reference lists and other sources searched or consulted to identify studies. Specify the date when each source was last searched or consulted.	4
Search strategy	7	Present the full search strategies for all databases, registers and websites, including any filters and limits used.	4-5<br>Tables S3-S5
Selection process	8	Specify the methods used to decide whether a study met the inclusion criteria of the review, including how many reviewers screened each record and each report retrieved, whether they worked independently, and if applicable, details of automation tools used in the process.	5
Data collection process	9	Specify the methods used to collect data from reports, including how many reviewers collected data from each report, whether they worked independently, any processes for obtaining or confirming data from study investigators, and if applicable, details of automation tools used in the process.	5
Data items	10a	List and define all outcomes for which data were sought. Specify whether all results that were compatible with each outcome domain in each study were sought (e.g. for all measures, time points, analyses), and if not, the methods used to decide which results to collect.	5-6
	10b	List and define all other variables for which data were sought (e.g. participant and intervention characteristics, funding sources). Describe any assumptions made about any missing or unclear information.	5
Study risk of bias assessment	11	Specify the methods used to assess risk of bias in the included studies, including details of the tool(s) used, how many reviewers assessed each study and whether they worked independently, and if applicable, details of automation tools used in the process.	6
Effect measures	12	Specify for each outcome the effect measure(s) (e.g. risk ratio, mean difference) used in the synthesis or presentation of results.	7
Synthesis methods	13a	Describe the processes used to decide which studies were eligible for each synthesis (e.g. tabulating the study intervention characteristics and comparing against the planned groups for each synthesis (item #5)).	6
	13b	Describe any methods required to prepare the data for presentation or synthesis, such as handling of missing summary statistics, or data conversions.	NA
	13c	Describe any methods used to tabulate or visually display results of individual studies and syntheses.	7
	13d	Describe any methods used to synthesize results and provide a rationale for the choice(s). If meta-analysis was performed, describe the model(s), method(s) to identify the presence and extent of statistical heterogeneity, and software package(s) used.	7
	13e	Describe any methods used to explore possible causes of heterogeneity among study results (e.g. subgroup analysis, meta-regression).	7

Section and Topic	Item #	Checklist item	Page
	13f	Describe any sensitivity analyses conducted to assess robustness of the synthesized results.	7
Reporting bias assessment	14	Describe any methods used to assess risk of bias due to missing results in a synthesis (arising from reporting biases).	7
Certainty assessment	15	Describe any methods used to assess certainty (or confidence) in the body of evidence for an outcome.	7
**RESULTS**			
Study selection	16a	Describe the results of the search and selection process, from the number of records identified in the search to the number of studies included in the review, ideally using a flow diagram.	8
	16b	Cite studies that might appear to meet the inclusion criteria, but which were excluded, and explain why they were excluded.	8
Study characteristics	17	Cite each included study and present its characteristics.	8-15<br>Tables 1 and 2
Risk of bias in studies	18	Present assessments of risk of bias for each included study.	9
Results of individual studies	19	For all outcomes, present, for each study: (a) summary statistics for each group (where appropriate) and (b) an effect estimate and its precision (e.g. confidence/credible interval), ideally using structured tables or plots.	9-15
Results of syntheses	20a	For each synthesis, briefly summarise the characteristics and risk of bias among contributing studies.	15-16
	20b	Present results of all statistical syntheses conducted. If meta-analysis was done, present for each the summary estimate and its precision (e.g. confidence/credible interval) and measures of statistical heterogeneity. If comparing groups, describe the direction of the effect.	16-20
	20c	Present results of all investigations of possible causes of heterogeneity among study results.	16-20
	20d	Present results of all sensitivity analyses conducted to assess the robustness of the synthesized results.	16-20
Reporting biases	21	Present assessments of risk of bias due to missing results (arising from reporting biases) for each synthesis assessed.	NA
Certainty of evidence	22	Present assessments of certainty (or confidence) in the body of evidence for each outcome assessed.	16-20
**DISCUSSION**			
Discussion	23a	Provide a general interpretation of the results in the context of other evidence.	21-24
	23b	Discuss any limitations of the evidence included in the review.	24-25
	23c	Discuss any limitations of the review processes used.	24-25
	23d	Discuss implications of the results for practice, policy, and future research.	26
**OTHER INFORMATION**			
Registration and protocol	24a	Provide registration information for the review, including register name and registration number, or state that the review was not registered.	28
	24b	Indicate where the review protocol can be accessed, or state that a protocol was not prepared.	28
	24c	Describe and explain any amendments to information provided at registration or in the protocol.	28
Support	25	Describe sources of financial or non-financial support for the review, and the role of the funders or sponsors in the review.	28
Competing interests	26	Declare any competing interests of review authors.	28
Availability of data, code and other materials	27	Report which of the following are publicly available and where they can be found: template data collection forms; data extracted from included studies; data used for all analyses; analytic code; any other materials used in the review.	28

Table S2. The PRISMA 2020 for Abstracts Checklist

Section and Topic	Item #	Checklist item	Reported (Yes/No)
**TITLE**			
Title	1	Identify the report as a systematic review.	Yes
**BACKGROUND**			
Objectives	2	Provide an explicit statement of the main objective(s) or question(s) the review addresses.	Yes
**METHODS**			
Eligibility criteria	3	Specify the inclusion and exclusion criteria for the review.	Yes
Information sources	4	Specify the information sources (e.g. databases, registers) used to identify studies and the date when each was last searched.	Yes
Risk of bias	5	Specify the methods used to assess risk of bias in the included studies.	Yes
Synthesis of results	6	Specify the methods used to present and synthesise results.	Yes
**RESULTS**			
Included studies	7	Give the total number of included studies and participants and summarise relevant characteristics of studies.	Yes
Synthesis of results	8	Present results for main outcomes, preferably indicating the number of included studies and participants for each. If meta-analysis was done, report the summary estimate and confidence/credible interval. If comparing groups, indicate the direction of the effect (i.e. which group is favoured).	Yes
**DISCUSSION**			
Limitations of evidence	9	Provide a brief summary of the limitations of the evidence included in the review (e.g. study risk of bias, inconsistency and imprecision).	NA
Interpretation	10	Provide a general interpretation of the results and important implications.	Yes
**OTHER**			
Funding	11	Specify the primary source of funding for the review.	NA
Registration	12	Provide the register name and registration number.	Yes

From: Page MJ, McKenzie JE, Bossuyt PM, Boutron I, Hoffmann TC, Mulrow CD, et al. The PRISMA 2020 statement: an updated guideline for reporting systematic reviews. BMJ 2021;372:n71. doi: 10.1136/bmj.n71

Table S3. Systematic search syntax for MEDLINE (via PubMed) with results as of December 12, 2023

No.	Syntax	No. of results
**1**	(Toll-like receptors [MeSH Terms] OR "toll-like receptors" [Title/Abstract] OR TLR [Title/Abstract] OR "toll-like receptor" [Title/Abstract] OR "Toll-like" [Title/Abstract] OR "Toll-Like receptor 4" [Title/Abstract] OR TLR4 [Title/Abstract] OR TLR-4 [Title/Abstract] OR "Toll-Like receptor 4" [MeSH Terms])	**73579**
**2**	(Schizophrenia [MeSH Terms] OR schizophrenia [Title/Abstract] OR psychotic [Title/Abstract] OR psychosis [Title/Abstract] OR psychotic disorders [MeSH Terms] OR "psychotic disorders" [Title/Abstract] OR "psychotic disorder" [Title/Abstract] OR schizophrenias [Title/Abstract] OR schizophrenic [Title/Abstract])	**228052**
**3**	#1 AND #2	**112**

Table S4. Systematic search syntax for Web of Science with results as of December 12, 2023

No.	Syntax	No. of results
**1**	(TS= (“Toll-like receptors”) OR TS= (“toll-like receptor”) OR TS= (TLR) OR TS= (“Toll-like”) OR TS= (“Toll-Like receptor 4”) OR TS= (TLR4) OR TS= (TLR-4))	**87616**
**2**	(TS= (Schizophrenia) OR TS= (psychotic) OR TS= (psychosis) OR TS= (“psychotic disorders”) OR TS= (“psychotic disorder”) OR TS= (schizophrenias) OR TS= (schizophrenic))	**266798**
**3**	#1 AND #2	**171**

Table S5. Systematic search syntax for Scopus with results as of December 12, 2023

No.	Syntax	No. of results
1	(INDEXTERMS("Toll-like receptors") OR TITLE-ABS("toll-like receptors") OR TITLE-ABS(TLR) OR TITLE-ABS("toll-like receptor") OR TITLE-ABS(Toll-like) OR TITLE-ABS("Toll-Like receptor 4") OR TITLE-ABS(TLR4) OR TITLE-ABS(TLR-4) OR INDEXTERMS("Toll-Like receptor 4"))	111233
2	(TITLE-ABS-KEY (Schizophrenia) OR TITLE-ABS-KEY (psychotic) OR TITLE-ABS-KEY (psychosis) OR TITLE-ABS-KEY ("psychotic disorders") OR TITLE-ABS-KEY ("psychotic disorder") OR TITLE-ABS-KEY (schizophrenias) OR TITLE-ABS-KEY (schizophrenic))	340059
3	#1 AND #2	339

Table S6. Studies identified in the literature search that proceeded to full-text screening[1]

Title	First author	Eligibility	Exclusion reason/ Extractors[2]
1. JNK signaling mediates aspects of maternal immune activation: importance of maternal genotype in relation to schizophrenia risk	R.L Openshow [1]	Exclude	Animal study
2. CNS Macrophages and Infant Infections	A. Oschwald [2]	Exclude	Review
3. Role of inflammation in epilepsy and neurobehavioral comorbidities: Implication for therapy	Y.N. Paudel [3]	Exclude	Review
4. The role of the gut microbiota in the pathophysiology of mental and neurological disorders	M.M. Pusceddu [4]	Exclude	Review
5. N-3 polyunsaturated fatty acids and clozapine abrogates poly I: C-induced immune alterations in primary hippocampal neurons	B.M.M. Ribeiro [5]	Exclude	In-vitro
6. Lack of Helios During Neural Development Induces Adult Schizophrenia-Like Behaviors Associated With Aberrant Levels of the TRIF-Recruiter Protein WDFY1	A. Sancho-Balsells [6]	Exclude	Animal study
7. Targeting the NLRP3 Inflammasome-Related Pathways via Tianeptine Treatment-Suppressed Microglia Polarization to the M1 Phenotype in Lipopolysaccharide-Stimulated Cultures	J. Slusarczyk [7]	Exclude	In-vitro
8. Importance of the immune system in mediating plasticity of the brain and behavior	L. Tian [8]	Exclude	Review
9. Evidence of microglial activation following exposure to serum from first-onset drug-naïve schizophrenia patients	G.F.V. Rees [9]	Exclude	levels of TLR were not discussed.
10. CD157 and Brain Immune System in (Patho)physiological Conditions: Focus on Brain Plasticity	O.L. Lopatina [10]	Exclude	Review
11. Neuroinflammation in the dorsolateral prefrontal cortex in elderly chronic schizophrenia	I. Lopez-Gonzalez [11]	Exclude	Post-mortem study
12. The Role of Immune Factors in Shaping Fetal Neurodevelopment	A. Lu-Culligan [12]	Exclude	Review
13. Differential regulation of the TLR4 signaling pathway in post-mortem prefrontal cortex and cerebellum in chronic schizophrenia: Relationship with SP transcription factors	K.S. MacDowell [13]	Exclude	Post-mortem study
14. Immunomodulatory effects of antipsychotic treatment on gene expression in first-episode psychosis	O. Mantere [14]	Exclude	Discussed psychosis, not schizophrenia specifically
15. Emerging role of innate B1 cells in the pathophysiology of autoimmune and neuroimmune diseases: Association with inflammation, oxidative and nitrosative stress and autoimmune responses	G. Morris [15]	Exclude	Review
16. Active psychosis and pro-inflammatory cytokines in first episode of psychosis	G. Pardo-de-Santayana [16]	Exclude	TLR was not discussed, cytokines were investigated
17. Anxiety-like behavior and microglial activation in the amygdala after acute neuroinflammation induced by microbial neuraminidase	A. Leon-Rodriguez [17]	Exclude	Animal study
18. Association between TLR2 polymorphisms (- 196-174 Ins/Del, R677W, R753Q, and P631H) and schizophrenia in a Tunisian population	Y. Aflouk [18]	Exclude	Levels of TLR were not discussed, TLR2 polymorphisms were only discussed
19. Association of complement component 4 with neuroimmune abnormalities in the subventricular zone in schizophrenia and autism spectrum disorders	T. C. M. Mou [19]	Exclude	No TLR was discussed, and complement component 4 was explained

[1] Row colors: gray: non-original articles; orange: animal studies; pink: studies not evaluating TLR4-related outcomes; purple: post-mortem studies, blue: evaluating outcomes in populations other than people with schizophrenia.
[2] Exclusion reason(s) is reported for the excluded studies. Name of data extractors and date of data extraction are reported for the included studies.

20. The association of inflammatory status and immunological parameters with single-nucleotide polymorphisms of cytokine and Toll-like receptor genes in patients with schizophrenia	I. K. Malashenkova [20]	Exclude	No levels of TLR were discussed, TLR polymorphisms were only discussed
21. Association of TLR-4 896A/G, TLR-4 1196C/T, and TLR-9 C/T polymorphism with schizophrenia in Indian Bengalee patient	J. Gurung [21]	Exclude	No levels of TLR were discussed, TLR polymorphisms were only discussed
22. Association of toll-like receptor 2 gene polymorphism (rs3804099) with susceptibility to Schizophrenia risk in the Dogra population of Jammu region, North India	I. Sharma [22]	Exclude	No levels of TLR were discussed, TLR polymorphisms were only discussed
23. Astrocyte Bioenergetics and Major Psychiatric Disorders	I. V. Maly [23]	Exclude	Book Series
24. Charting the proteome landscape in major psychiatric disorders: From biomarkers to biological pathways towards drug discovery	Fern, B. S. es [24]	Exclude	systematic review and comprehensive *in silico* analysis
25. Cognitive impairment in psychiatric diseases: Biomarkers of diagnosis, treatment, and prevention	Y. Wang [25]	Exclude	Review
26. Consequences of Viral Infection and Cytokine Production During Pregnancy on Brain Development in Offspring	D. Elgueta [26]	Exclude	Review
27. Differential Effects of Toll-Like Receptor Activation and Differential Mediation by MAP Kinases of Immune Responses in Microglial Cells	J. Kwon, C. Arsenis [27]	Exclude	In-vitro
28. Dysfunctional monocytic toll-like receptor 4 signaling pathway and cognitive deficits in chronic schizophrenia patients with tardive dyskinesia	N. Li [28]	Include	* Data extractors (February 2024): M.J., R.S., S.B.<br>* Conflicts: M.A. and M.J.
29. Early life Adversity, functional connectivity and cognitive performance in Schizophrenia: The mediating role of IL-6	S. King [29]	Exclude	No levels of TLR were discussed
30. Editorial: Cognitive Dysfunctions in Psychiatric Disorders: Brain-Immune Interaction Mechanisms and Integrative Therapeutic Approaches	H. Xu [30]	Exclude	Editorial
31. Editorial: The inflammation markers in schizophrenia and bipolar disorder: Do we have promising results?	A. Arslan [31]	Exclude	Editorial
32. Environmental Risk Factors for Schizophrenia and Bipolar Disorder and Their Relationship to Genetic Risk: Current Knowledge and Future Directions	N. Robinson [32]	Exclude	Review
33. First Episode Psychosis and Schizophrenia Are Systemic Neuro-Immune Disorders Triggered by a Biotic Stimulus in Individuals with Reduced Immune Regulation and Neuroprotection	M. Maes [33]	Exclude	Secondary analysis, no data on TLR
34. Gut microbiota in psychiatric disorders: Better understanding or more complexity to be resolved?	B. Misiak [34]	Exclude	Editorial
35. Human endogenous retrovirus W family envelope protein (HERV-W env) facilitates the production of TNF-α and IL-10 by inhibiting MyD88s in glial cells	X. Wang [35]	Exclude	In-vitro
36. Identifying crucial biomarkers in peripheral blood of schizophrenia and screening therapeutic agents by comprehensive bioinformatics analysis	M. Xie [36]	Exclude	Secondary analysis, no distinct data on the levels of TLR
37. Immune System Abnormalities in Schizophrenia: An Integrative View and Translational Perspectives	E. Ermakov [37]	Exclude	Review
38. Impact of SARS-CoV-2 on Host Factors Involved in Mental Disorders	R. Rhoades [38]	Exclude	Review
39. Influenza Virus Infection during Pregnancy as a Trigger of Acute and Chronic Complications	O. Oseghale [39]	Exclude	Review
40. Involvement of TCF7L2 in generation of morphine-induced antinociceptive tolerance and hyperalgesia by modulating TLR4/ NF-κB/NLRP3 in microglia	J. Chen [40]	Exclude	Animal study

41. A loss of mature microglial markers without immune activation in schizophrenia	G. J. L. J. Snijders [41]	Exclude	Review
42. Maternal inflammation and its ramifications on fetal neurodevelopment	H.K. Kwon [42]	Exclude	Review
43. Modulatory Effect of Gut Microbiota on the Gut-Brain, Gut-Bone Axes, and the Impact of Cannabinoids	I. Ibrahim [43]	Exclude	Review
44. Negative Regulation of the IL-1 System by IL-1R2 and IL-1R8: Relevance in Pathophysiology and Disease	D. Supino [44]	Exclude	Review
45. Neurobiological Highlights of Cognitive Impairment in Psychiatric Disorders	A. Morozova [45]	Exclude	Review
46. Peripheral NF-κB dysregulation in people with schizophrenia drives inflammation: putative anti-inflammatory functions of NF-κB kinases	C.E. Murphy [46]	Exclude	Evaluating TLR4 gene expression in peripheral blood leukocytes (not inclusively in PBMC)
47. A Possible Causal Involvement of Neuroinflammatory, Purinergic P2X7 Receptors in Psychiatric Disorders	Y. Zhang [47]	Exclude	Review
48. Potential Neurocognitive Symptoms Due to Respiratory Syncytial Virus Infection	C.A. Andrade [48]	Exclude	Review
49. Psychosis symptoms following aberrant immunity in the brain	A. Ozaki [49]	Exclude	Editorial
50. The relationship between TLR4/NF-κB/IL-1β signaling, cognitive impairment, and white-matter integrity in patients with stable chronic schizophrenia	H. Li [50]	Included	* Data extractors (February 2024): M.J., R.S., S.B.<br>* Conflicts: M.A. and M.J.
51. The role of the gut microbiome in the development of schizophrenia	J.R. Kelly [51]	Exclude	Review
52. Schizophrenia Hypothesis: Autonomic Nervous System Dysregulation of Fetal and Adult Immune Tolerance	T. Carnac [52]	Exclude	Review
53. Targeting s100b protein as a surrogate biomarker and its role in various neurological disorders	U. Langeh [53]	Exclude	Review
54. TOLL-LIKE RECEPTOR (TLR) PATHWAY EXPRESSION IN DORSOLATERAL PREFRONTAL (BA46) AND ORBITOFRONTAL (BA11) CORTICES IN SCHIZOPHRENIA AND MOOD DISORDER	T. Ketharanathan [54]	Exclude	Post-mortem study on schizophrenia
55. Toll-Like Receptor 4 Gene Polymorphisms and Susceptibility to Schizophrenia: A Case-Control Study	M. Mostafa [55]	Exclude	No levels of TLR were discussed, TLR polymorphisms were only discussed
56. Virus-Induced Maternal Immune Activation as an Environmental Factor in the Etiology of Autism and Schizophrenia	A. Massrali [56]	Exclude	Review
57. Antipsychotics influence Toll-like receptor (TLR) expression and its relationship with cognitive functions in schizophrenia	S. Keri [57]	Include	* Data extractors (February 2024): M.J., R.S., S.B.<br>* Conflicts: M.A. and M.J.
58. Uniting the neurodevelopmental and immunological hypotheses: Neuregulin 1 receptor ErbB and Toll-like receptor activation in first-episode schizophrenia	S. Keri [58]	Exclude	Erratum
59. The expression of toll-like receptors in peripheral blood mononuclear cells is altered in schizophrenia	E. Kozlowska [59]	Include	* Data extractors (February 2024): M.J., R.S., S.B.<br>* Conflicts: M.A. and M.J.
60. Oxidative stress and mitochondrial dysfunction in human diseases: Pathophysiology, predictive biomarkers, therapeutic	C.J. Li [60]	Exclude	Editorial
61. TLR4 (Toll-like receptor-4) expression and frontal-cingulate volumes in schizophrenia	H. Li [61]	Exclude	Non-English

62. The TMEM106B FTLD-protective variant, rs1990621, is also associated with increased neuronal proportion	Z. Li [62]	Exclude	No TLRs were measured
63. Maternal Murine Cytomegalovirus Infection during Pregnancy Up-regulates the Gene Expression of Toll-like Receptor 2 and 4 in Placenta	Y. Liao [63]	Exclude	Animal study
64. Periodontal pathogens and neuropsychiatric health	A. Wadhawan [64]	Exclude	Review
65. Human endogenous retroviral envelope protein Syncytin-1 and inflammatory abnormalities in neuropsychological diseases	X. Wang [65]	Exclude	Review
66. Syncytin-1, an endogenous retroviral protein, triggers the activation of CRP via TLR3 signal cascade in glial cells	X. Wang [66]	Exclude	In-vitro
67. Longitudinal Analyses of Blood Transcriptome During Conversion to Psychosis	B. Chaumette [67]	Exclude	No TLRs were measured
68. More dampened monocytic Toll-like receptor 4 response to lipopolysaccharide and its association with cognitive function in Chinese Han first-episode patients with schizophrenia	S. Chen [68]	Include	* Data extractors (February 2024): M.J., R.S., S.B.<br>* Conflicts: M.A. and M.J.
69. Natural Catalytic IgGs Hydrolyzing Histones in Schizophrenia: Are They the Link between Humoral Immunity and Inflammation?	E.A. Ermakov [69]	Exclude	No TLRs were measured
70. Neural cell responses upon exposure to human endogenous retroviruses	J. Gruchot [70]	Exclude	Review
71. Neuroimmune biomarkers in mental illness	J.W. Herron [71]	Exclude	Review
72. Microbiota-immune system interactions in human neurological disorders	Q. Huang [72]	Exclude	Review
73. Expression and Functionality Study of 9 Toll-Like Receptors in 33 Drug-Naive Non-Affective First Episode Psychosis Individuals: A 3-Month Study	M. Juncal-Ruiz [73]	Exclude	Discussed psychosis, not schizophrenia specifically
74. Cross talk: The microbiota and neurodevelopmental disorders	J.R. Kelly [74]	Exclude	Review
75. Erratum: Uniting the neurodevelopmental and immunological hypotheses: Neuregulin 1 receptor ErbB and Toll-like receptor activation in first-episode schizophrenia	S. Keri [75]	Exclude	Erratum
76. Uniting the neurodevelopmental and immunological hypotheses: Neuregulin 1 receptor ErbB and Toll-like receptor activation in first-episode schizophrenia	S. Keri [76]	Include	* Data extractors (February 2024): M.J., R.S., S.B.<br>* Conflicts: M.A. and M.J.
77. The role of cytokines in mediating effects of prenatal infection on the fetus: implications for schizophrenia	H. Ashdown [77]	Exclude	Review
78. Neonatal polyI:C treatment in mice results in schizophrenia-like behavioral and neurochemical abnormalities in adulthood	D. Ibi [78]	Exclude	Animal study
79. Expression of anti-cardiolipin antibodies and inflammatory associated factors in patients with schizophrenia	SH. Chang [79]	Include	* Data extractors (February 2024): M.J., R.S., S.B.<br>* Conflicts: M.A. and M.J.
80. ALTERED TOLL-LIKE RECEPTOR RESPONSES IN SCHIZOPHRENIA AND BIPOLAR DISORDER PATIENTS	D.P. McKernan [80]	Exclude	Review
81. Enhanced peripheral toll-like receptor responses in psychosis: further evidence of a pro-inflammatory phenotype	D. P. McKernan [81]	Exclude	TLR-4 was not measured
82. Cellular and Molecular Mechanisms of Autoimmune Disease	B. Bolon [82]	Exclude	Review
83. Monocytic HLA DR antigens in schizophrenic patients	D. Krause [83]	Exclude	TLR-4 was not measured
84. Toll-like receptor 2 polymorphisms are associated with poor concentration in schizophrenia patients in a Korean population	S. M. Lee [84]	Exclude	TLR-4 was not measured

85. Impaired monocyte activation in schizophrenia	N. Müller [85]	Include	* Data extractors (February 2024): M.J., R.S., S.B.<br>* Conflicts: M.A. and M.J.
86. Possible involvement of TLRs and hemichannels in stress-induced CNS dysfunction via mastocytes, and glia activation	A. Aguirre [86]	Exclude	Review
87. Pathophysiology of bacterial infection of the central nervous system and its putative role in the pathogenesis of behavioral changes	T. Barichello [87]	Exclude	Review
88. Oligodendroglial Alterations and the Role of Microglia in White Matter Injury: Relevance to Schizophrenia	L. J. Chew [88]	Exclude	Review
89. Neuroinflammation in mood disorders: Mechanisms and drug targets	A. T. Hopper [89]	Exclude	Review
90. Association between genetic polymorphisms of Toll-like receptor 2 (TLR2) and schizophrenia in the Korean population	W. S. Kang [90]	Exclude	TLR-4 was not measured
91. TLR7 Negatively Regulates Dendrite Outgrowth through the Myd88-c-Fos-IL-6 Pathway	H. Y. Liu [91]	Exclude	Animal study and in-vitro
92. Role of the Toll Like receptor (TLR) radical cycle in chronic inflammation: possible treatments targeting the TLR4 pathway	K. Lucas [92]	Exclude	Review
93. Innate immunity and neuroinflammation	A. Shastri [93]	Exclude	Review
94. Intermittent or sustained systemic inflammation and the preterm brain	O. Dammann [94]	Exclude	Review
95. Sarm1 deficiency impairs synaptic function and leads to behavioral deficits, which can be ameliorated by an mGluR allosteric modulator	C. W. Lin [95]	Exclude	No TLR were measured
96. Effects of Genetic and Environmental Factors on Neuropsychological Development	T. Nagai [96]	Exclude	Review
97. Influenza vaccination during early pregnancy contributes to neurogenesis and behavioral function in offspring	Y. C. Xia [97]	Exclude	Animal study
98. Protein-interaction-network-based analysis for genome-wide association analysis of schizophrenia in Han Chinese population	H. Yu [98]	Exclude	No TLR were measured
99. A molecular pathway analysis informs the genetic background at risk for schizophrenia	C. Crisafulli [99]	Exclude	This article discussed molecular pathways and no TLR were measured.
100. Innate immune response is differentially dysregulated between bipolar disease and schizophrenia	A. de Baumont [100]	Exclude	No TLR were measured
101. Psychedelics and Immunomodulation: Novel Approaches and Therapeutic Opportunities	A. Szabo [101]	Exclude	Review
102. Commentary: Maternal immune activation evoked by polyinosinic: Polycytidylic acid does not evoke microglial cell activation in the embryo	H. G. Bernstein [102]	Exclude	Commentary
103. The Microbiota and Gut-Brain Axis: Contributions to the Immunopathogenesis of Schizophrenia	J. R. Caso [103]	Exclude	Review
104. Interactions between the gut microbiome and the central nervous system and their role in schizophrenia, bipolar disorder and depression	A. A. Chrobak [104]	Exclude	Review
105. Innate immune receptor Toll-like receptor 4 signaling in neuropsychiatric diseases	B. García Bueno [105]	Exclude	Review
106. Evidence of activation of the Toll-like receptor-4 proinflammatory pathway in patients with schizophrenia	B. García-Bueno [106]	Exclude	Post-mortem study
107. Activation of maternal toll-like receptor-2 causes social deficits and memory impairment in female offspring	Y. Lee [107]	Exclude	Animal study
108. Tryptophan Metabolism Along the Kynurenine Pathway Downstream of Toll-like Receptor Stimulation in Peripheral Monocytes	F. Orhan [108]	Exclude	Healthy subjects were recruited in this study

109. Autoimmune diseases, gastrointestinal disorders and the microbiome in schizophrenia: more than a gut feeling	E. G. Severance [109]	Exclude	Review
110. Experimental brain ischemic preconditioning: A concept to putative targets	A. Sharma [110]	Exclude	Review
111. Erratum: Uniting the neurodevelopmental and immunological hypotheses: Neuregulin 1 receptor ERBB and Toll-like receptor activation in first-episode schizophrenia	S. Keri [76]	Exclude	Erratum (duplicate)
112. Evidence for Decreased Density of Calretinin-Immunopositive Neurons in the Caudate Nucleus in Patients With Schizophrenia	I. Adorjan [111]	Exclude	No TLRs were measured
113. Microbiome, inflammation, epigenetic alterations, and mental diseases	R. Alam [112]	Exclude	Review
114. Sex-dependent effects of perinatal inflammation on the brain: Implication for neuro-psychiatric disorders	M. Ardalan [113]	Exclude	Review
115. Pattern of expression of Toll like receptor (TLR)-3 and -4 genes in drug-naïve and antipsychotic treated patients diagnosed with schizophrenia	R. Balaji [114]	Include	* Data extractors (February 2024): M.J., R.S., S.B.<br>* Conflicts: M.A. and M.J.
116. Traumagenics: At the intersect of childhood trauma, immunity and psychosis	KA. Chase [115]	Include	* Data extractors (February 2024): M.J., R.S., S.B.<br>* Conflicts: M.A. and M.J.
117. From probiotics to psychobiotics - the gut-brain axis in psychiatric disorders	A. Zagorska [116]	Exclude	Review
118. TLR/mTOR inflammatory signaling pathway: novel insight for the treatment of schizophrenia	N. A. Lashgari [117]	Exclude	Review
119. The Emerging Role of Toll-Like Receptor-Mediated Neuroinflammatory Signals in Psychiatric Disorders and Acquired Epilepsy	A. Chaudhary [118]	Exclude	Review
120. Association between genetic variants of TLR2, TLR4, TLR9 and schizophrenia	F. Ayari [119]	Exclude	Non-English
121. Aberrant Histone Modification of TNFAIP3, TLR4, TNIP2, miR-146a, and miR-155 in Major Depressive Disorder	C. C. Tseng [120]	Exclude	Discussed just major depressive disorder
122. Toll-Like Receptor (TLR) 1, 2, and 6 Gene Polymorphisms Support Evidence of Innate Immune Factors in Schizophrenia	C. E. Sotelo-Ramírez [121]	Exclude	TLR-4 was not measured
123. Increased proportions of circulating proinflammatory monocytes and macrophages expressing toll-like receptor 4 in individuals with schizophrenia and bipolar disorder receiving medication.	SY. Tsai [122]	Include	* Data extractors (February 2024): M.J., R.S., S.B.<br>* Conflicts: M.A. and M.J.
124. Counting the Toll of Inflammation on Schizophrenia-A Potential Role for Toll-like Receptors	S. R. Patlola [123]	Exclude	Review
125. Whole Genome Sequencing Revealed Inherited Rare Oligogenic Variants Contributing to Schizophrenia and Major Depressive Disorder in Two Families	I. H. Chung [124]	Exclude	Discussed just major depressive disorder

Table S7. Risk of bias evaluation, the Joanna Briggs Institute (JBI) Critical Appraisal tool

Questions	Chang 2011	Müller 2012	Kéri 2016	Kéri 2017	Chen 2019	Balaji 2019	Chase 2019	Kozłowska 2019	H. Li 2022	N. Li 2022	Tsai 2023
**1.** Were the groups comparable other than the presence of disease in cases or the absence of disease in controls?	age: n<br>sex: n<br>BMI: NA<br>smoke: NA	age: y<br>sex: y<br>BMI: y<br>smoking: n	age: y<br>sex: y<br>BMI: y<br>smoking: y	age: y<br>sex: y<br>BMI: y<br>smoking: y	age: y<br>sex: y<br>BMI: y<br>smoking: y	age: n<br>sex: y<br>BMI: NA<br>smoking: NA	age: y<br>sex: y<br>BMI: n<br>smoking: y	age: y<br>sex: y<br>BMI: n<br>smoking: n	age: y<br>sex: y<br>BMI: NA<br>smoking: NA	age: y<br>sex: y<br>BMI: y<br>smoking: NA	age: y<br>sex: y<br>BMI: y<br>smoking: y
**2.** Were cases and controls matched appropriately?		Individual matching age, sex, ethnicity	Individual matching: age, sex, education	Individual matching: age, sex, education				Individual matching: metabolic parameters			Individual matching: age
**3.** Were the same criteria used for identification of cases and controls?											
**4.** Was exposure measured in a standard, valid and reliable way?											
**5.** Was exposure measured in the same way for cases and controls?											
**6.** Were confounding factors identified?											
**7.** Were strategies to deal with confounding factors stated?											
**8.** Were outcomes assessed in a standard, valid and reliable way for cases and controls?											
**9.** Was the exposure period of interest long enough to be meaningful?											
**10.** Was appropriate statistical analysis used?											

Table 2 Quality assessment chart (YES, NO, Unclear, NA)

Abbreviations: y: yes; n: no; NA: not available

Figure S1. Galbraith plots

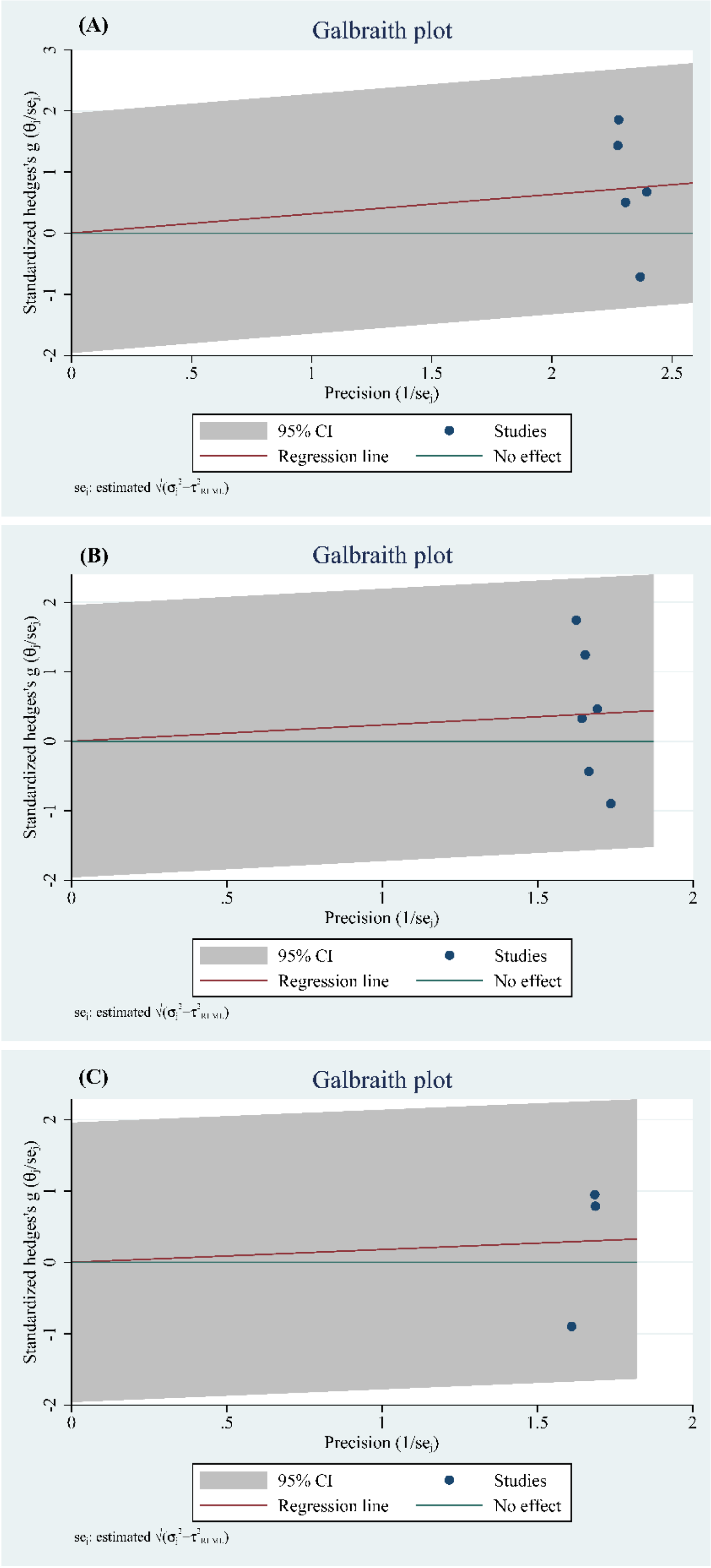

Figure S1. **A)** Basal surface monocytic TLR4 density, **B)** Basal percentage of TLR4-expressing monocytes, and **C)** Basal TLR4 gene expression in PBMCs

Figure S2. Funnel Plots

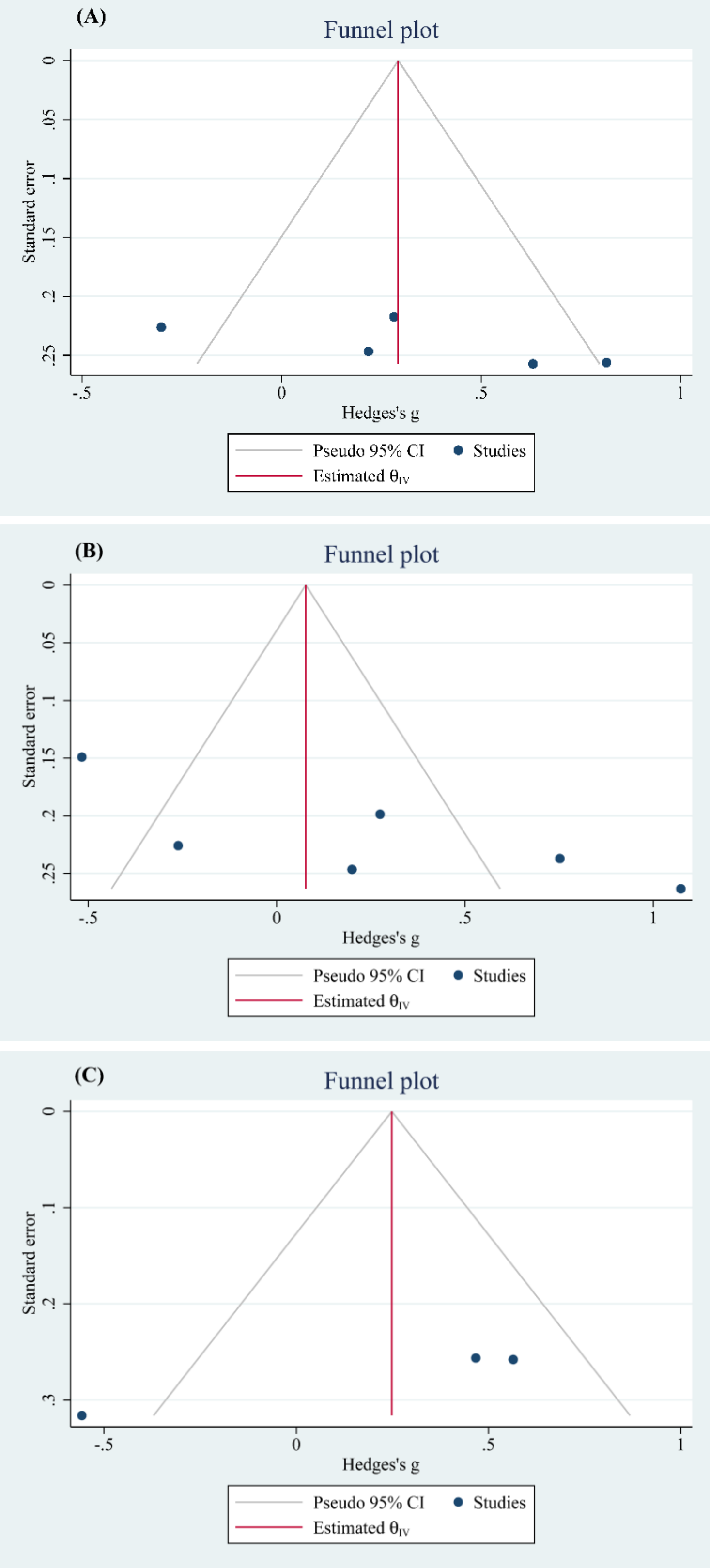

Figure S2. **A)** Basal surface monocytic TLR4 density, **B)** Basal percentage of TLR4-expressing monocytes, and **C)** Basal TLR4 gene expression in PBMCs

References

1. Openshaw RL, Kwon J, McColl A, et al. JNK signalling mediates aspects of maternal immune activation: importance of maternal genotype in relation to schizophrenia risk. *JOURNAL OF NEUROINFLAMMATION*. 2019-1-28 2019;16doi:doi:10.1186/s12974-019-1408-5
2. Oschwald A, Petry P, Kierdorf K, Erny D. CNS Macrophages and Infant Infections. *Frontiers in Immunology*. 2020 2020;11doi:doi:10.3389/fimmu.2020.02123
3. Paudel YN, Shaikh MF, Shah S, Kumari Y, Othman I. Role of inflammation in epilepsy and neurobehavioral comorbidities: Implication for therapy. *European Journal of Pharmacology*. 2018 2018;837:145-155. doi:doi:10.1016/j.ejphar.2018.08.020
4. Pusceddu MM, Del Bas JM. The role of the gut microbiota in the pathophysiology of mental and neurological disorders. *Psychiatric Genetics*. 2020 2020;30(4):87-100. doi:doi:10.1097/YPG.0000000000000255
5. Ribeiro BMM, Chaves Filho AJM, Costa DVDS, et al. N-3 polyunsaturated fatty acids and clozapine abrogates poly I: C-induced immune alterations in primary hippocampal neurons. *Progress in Neuro-Psychopharmacology and Biological Psychiatry*. 2019 2019;90:186-196. doi:doi:10.1016/j.pnpbp.2018.11.022
6. Sancho-Balsells A, Brito V, Fernández B, et al. Lack of Helios During Neural Development Induces Adult Schizophrenia-Like Behaviors Associated With Aberrant Levels of the TRIF-Recruiter Protein WDFY1. *Frontiers in Cellular Neuroscience*. 2020 2020;14doi:doi:10.3389/fncel.2020.00093
7. Slusarczyk J, Trojan E, Glombik K, et al. Targeting the NLRP3 Inflammasome-Related Pathways via Tianeptine Treatment-Suppressed Microglia Polarization to the M1 Phenotype in Lipopolysaccharide-Stimulated Cultures. *INTERNATIONAL JOURNAL OF MOLECULAR SCIENCES*. 2018-7 2018;19(7)doi:doi:10.3390/ijms19071965
8. Tian L, Tremblay ME. Importance of the immune system in mediating plasticity of the brain and behavior. *Progress in Neuro-Psychopharmacology and Biological Psychiatry*. 2017 2017;79:1-2. doi:doi:10.1016/j.pnpbp.2017.06.004
9. van Rees GF, Lago SG, Cox DA, et al. Evidence of microglial activation following exposure to serum from first-onset drug-naïve schizophrenia patients. *Brain, Behavior, and Immunity*. 2018 2018;67:364-373. doi:doi:10.1016/j.bbi.2017.10.003
10. Lopatina OL, Komleva YK, Malinovskaya NA, Panina YA, Morgun AV, Salmina AB. CD157 and Brain Immune System in (Patho)physiological Conditions: Focus on Brain Plasticity. *Frontiers in Immunology*. 2020 2020;11doi:doi:10.3389/fimmu.2020.585294
11. López-González I, Pinacho R, Vila È, Escanilla A, Ferrer I, Ramos B. Neuroinflammation in the dorsolateral prefrontal cortex in elderly chronic schizophrenia. *Eur Neuropsychopharmacol*. 2019-3 2019;29(3):384-396. doi:doi:10.1016/j.euroneuro.2018.12.011
12. Lu-Culligan A, Iwasaki A. *The Role of Immune Factors in Shaping Fetal Neurodevelopment*. 2020. https://www.scopus.com/inward/record.uri?eid=2-s2.0-85092681002&doi=10.1146%2fannurev-cellbio-021120-033518&partnerID=40&md5=13a19346af9614f463e86f141dd4278b
13. MacDowell KS, Pinacho R, Leza JC, Costa J, Ramos B, García-Bueno B. Differential regulation of the TLR4 signalling pathway in post-mortem prefrontal cortex and cerebellum in chronic schizophrenia: Relationship with SP transcription factors. *Prog Neuropsychopharmacol Biol Psychiatry*. 2017-10-3 2017;79:481-492. doi:doi:10.1016/j.pnpbp.2017.08.005
14. Mantere O, Trontti K, García-González J, et al. Immunomodulatory effects of antipsychotic treatment on gene expression in first-episode psychosis. *Journal of Psychiatric Research*. 2019 2019;109:18-26. doi:doi:10.1016/j.jpsychires.2018.11.008
15. Morris G, Puri BK, Olive L, Carvalho AF, Berk M, Maes M. Emerging role of innate B1 cells in the pathophysiology of autoimmune and neuroimmune diseases: Association with inflammation, oxidative and nitrosative stress and autoimmune responses. *Pharmacol Res*. 2019-10 2019;148:104408. doi:doi:10.1016/j.phrs.2019.104408
16. Pardo-de-Santayana G, Juncal-Ruiz M, Vázquez-Bourgon J, et al. Active psychosis and pro-inflammatory cytokines in first-episode of psychosis. *J Psychiatr Res*. 2021-2 2021;134:150-157. doi:doi:10.1016/j.jpsychires.2020.12.060
17. Leon-Rodriguez A, Fern, ez-Arjona MD, Grondona JM, Pedraza C, Lopez-Avalos MD. Anxiety-like behavior and microglial activation in the amygdala after acute neuroinflammation induced by microbial neuraminidase. *SCIENTIFIC REPORTS*. 2022-7-8 2022;12(1)doi:doi:10.1038/s41598-022-15617-5
18. Aflouk Y, Inoubli O, Saoud H, Zaafrane F, Gaha L, Bel Hadj Jrad B. Association between TLR2 polymorphisms (- 196-174 Ins/Del, R677W, R753Q, and P631H) and schizophrenia in a Tunisian population. *Immunol Res*. 2021-12 2021;69(6):541-552. doi:doi:10.1007/s12026-021-09238-9
19. Mou TCM, Lane MV, Irel, et al. Association of complement component 4 with neuroimmune abnormalities in the subventricular zone in schizophrenia and autism spectrum disorders. *Neurobiology of Disease*. 2022 2022;173doi:doi:10.1016/j.nbd.2022.105840

20. Malashenkova IK, Ushakov VL, Krynskiy SA, et al. The association of inflammatory status and immunological parameters with single-nucleotide polymorphisms of cytokine and Toll-like receptor genes in patients with schizophrenia. *Research Results in Biomedicine*. 2022 2022;8(2):148-163. doi:doi:10.18413/2658-6533-2022-8-2-0-2
21. Gurung J, Bera NK, Lama M, Singh B. Association of TLR-4 896A/G, TLR-4 1196C/T, and TLR-9 C/T polymorphism with schizophrenia in Indian Bengalee patient. *Indian J Psychiatry*. 2022-11 2022;64(6):579-587. doi:doi:10.4103/indianjpsychiatry.indianjpsychiatry_263_22
22. Sharma I, Priya I, Sharma S, et al. Association of toll-like receptor 2 gene polymorphism (rs3804099) with susceptibility to Schizophrenia risk in the Dogra population of Jammu region, North India. *European Journal of Psychiatry*. 2022 2022;36(2):106-113. doi:doi:10.1016/j.ejpsy.2022.02.001
23. Maly IV, Morales MJ, Pletnikov MV. *Astrocyte Bioenergetics and Major Psychiatric Disorders*. 2021. https://www.scopus.com/inward/record.uri?eid=2-s2.0-85121230075&doi=10.1007%2f978-3-030-77375-5_9&partnerID=40&md5=ee7c641acf8feb6e569c64fd85721baf
24. Fern, es BS, Dai Y, Jia P, Zhao Z. Charting the proteome landscape in major psychiatric disorders: From biomarkers to biological pathways towards drug discovery. *Eur Neuropsychopharmacol*. 2022-8 2022;61:43-59. doi:doi:10.1016/j.euroneuro.2022.06.001
25. Wang Y, Meng W, Liu Z, An Q, Hu X. Cognitive impairment in psychiatric diseases: Biomarkers of diagnosis, treatment, and prevention. *Frontiers in Cellular Neuroscience*. 2022 2022;16doi:doi:10.3389/fncel.2022.1046692
26. Elgueta D, Murgas P, Riquelme E, Yang G, Cancino GI. Consequences of Viral Infection and Cytokine Production During Pregnancy on Brain Development in Offspring. *Frontiers in Immunology*. 2022 2022;13doi:doi:10.3389/fimmu.2022.816619
27. Kwon J, Arsenis C, Suessmilch M, McColl A, Cavanagh J, Morris BJ. Differential Effects of Toll-Like Receptor Activation and Differential Mediation by MAP Kinases of Immune Responses in Microglial Cells. *CELLULAR AND MOLECULAR NEUROBIOLOGY*. 2022-11 2022;42(8):2655-2671. doi:doi:10.1007/s10571-021-01127-x
28. Li N, Li YL, Huang JC, et al. Dysfunctional monocytic toll-like receptor 4 signaling pathway and cognitive deficits in chronic schizophrenia patients with tardive dyskinesia. *NEUROSCIENCE LETTERS*. 2022-4-23 2022;777doi:doi:10.1016/j.neulet.2022.136581
29. King S, Holleran L, Mothersill D, et al. Early life Adversity, functional connectivity and cognitive performance in Schizophrenia: The mediating role of IL-6. *Brain Behav Immun*. 2021-11 2021;98:388-396. doi:doi:10.1016/j.bbi.2021.06.016
30. Xu H, Wang W, Ellenbroek B, You Z. Editorial: Cognitive Dysfunctions in Psychiatric Disorders: Brain-Immune Interaction Mechanisms and Integrative Therapeutic Approaches. *Frontiers in Integrative Neuroscience*. 2021 2021;15doi:doi:10.3389/fnint.2021.649425
31. Arslan A, Aydin O, Çökmüş FP. Editorial: The inflammation markers in schizophrenia and bipolar disorder: Do we have promising results? *Frontiers in Psychiatry*. 2023 2023;13doi:doi:10.3389/fpsyt.2022.1128355
32. Robinson N, Bergen SE. Environmental Risk Factors for Schizophrenia and Bipolar Disorder and Their Relationship to Genetic Risk: Current Knowledge and Future Directions. *Frontiers in Genetics*. 2021 2021;12doi:doi:10.3389/fgene.2021.686666
33. Maes M, Plaimas K, Suratanee A, Noto C, Kanchanatawan B. First Episode Psychosis and Schizophrenia Are Systemic Neuro-Immune Disorders Triggered by a Biotic Stimulus in Individuals with Reduced Immune Regulation and Neuroprotection. *Cells*. 2021-10-28 2021;10(11)doi:doi:10.3390/cells10112929
34. Misiak B, Samochowiec J, Marlicz W, Łoniewski I. Gut microbiota in psychiatric disorders: Better understanding or more complexity to be resolved? *Progress in Neuro-Psychopharmacology and Biological Psychiatry*. 2021 2021;110doi:doi:10.1016/j.pnpbp.2021.110302
35. Wang X, Wu X, Huang J, Li H, Yan Q, Zhu F. Human endogenous retrovirus W family envelope protein (HERV-W env) facilitates the production of TNF-α and IL-10 by inhibiting MyD88s in glial cells. *Arch Virol*. 2021-4 2021;166(4):1035-1045. doi:doi:10.1007/s00705-020-04933-8
36. Xie M, Li Z, Li X, et al. Identifying crucial biomarkers in peripheral blood of schizophrenia and screening therapeutic agents by comprehensive bioinformatics analysis. *Journal of Psychiatric Research*. 2022 2022;152:86-96. doi:doi:10.1016/j.jpsychires.2022.06.007
37. Ermakov EA, Melamud MM, Buneva VN, Ivanova SA. Immune System Abnormalities in Schizophrenia: An Integrative View and Translational Perspectives. *Frontiers in Psychiatry*. 2022 2022;13doi:doi:10.3389/fpsyt.2022.880568
38. Rhoades R, Solomon S, Johnson C, Teng S. Impact of SARS-CoV-2 on Host Factors Involved in Mental Disorders. *Frontiers in Microbiology*. 2022 2022;13doi:doi:10.3389/fmicb.2022.845559
39. Oseghale O, Vlahos R, O'Leary JJ, et al. Influenza Virus Infection during Pregnancy as a Trigger of Acute and Chronic Complications. *Viruses*. 2022 2022;14(12)doi:doi:10.3390/v14122729

40. Chen J, Wang G, Sun T, Ma C, Huo X, Kong Y. Involvement of TCF7L2 in generation of morphine-induced antinociceptive tolerance and hyperalgesia by modulating TLR4/ NF-κB/NLRP3 in microglia. *Toxicology and Applied Pharmacology*. 2021 2021;416doi:doi:10.1016/j.taap.2021.115458
41. Snijders GJLJ, van Zuiden W, Sneeboer MAM, et al. A loss of mature microglial markers without immune activation in schizophrenia. *GLIA*. 2021 2021;69(5):1251-1267. doi:doi:10.1002/glia.23962
42. Kwon HK, Choi GB, Huh JR. Maternal inflammation and its ramifications on fetal neurodevelopment. *Trends in Immunology*. 2022 2022;43(3):230-244. doi:doi:10.1016/j.it.2022.01.007
43. Ibrahim I, Syamala S, Ayariga JA, et al. Modulatory Effect of Gut Microbiota on the Gut-Brain, Gut-Bone Axes, and the Impact of Cannabinoids. *Metabolites*. 2022 2022;12(12)doi:doi:10.3390/metabo12121247
44. Supino D, Minute L, Mariancini A, et al. Negative Regulation of the IL-1 System by IL-1R2 and IL-1R8: Relevance in Pathophysiology and Disease. *Frontiers in Immunology*. 2022 2022;13doi:doi:10.3389/fimmu.2022.804641
45. Morozova A, Zorkina Y, Abramova O, et al. Neurobiological Highlights of Cognitive Impairment in Psychiatric Disorders. *International Journal of Molecular Sciences*. 2022 2022;23(3)doi:doi:10.3390/ijms23031217
46. Murphy CE, Walker AK, O'Donnell M, et al. Peripheral NF-κB dysregulation in people with schizophrenia drives inflammation: putative anti-inflammatory functions of NF-κB kinases. *Translational Psychiatry*. 2022 2022;12(1)doi:doi:10.1038/s41398-021-01764-2
47. Zhang Y, Yin HY, Rubini P, Tang Y, Illes P. A Possible Causal Involvement of Neuroinflammatory, Purinergic P2X7 Receptors in Psychiatric Disorders. *Curr Neuropharmacol*. 2022 2022;20(11):2142-2155. doi:doi:10.2174/1570159x20666220302152400
48. Andrade CA, Kalergis AM, Bohmwald K. Potential Neurocognitive Symptoms Due to Respiratory Syncytial Virus Infection. *Pathogens*. 2022 2022;11(1)doi:doi:10.3390/pathogens11010047
49. Ozaki A, Yamawaki Y, Ohtsuki G. Psychosis symptoms following aberrant immunity in the brain. *Neural Regeneration Research*. 2021 2021;16(3):512-513. doi:doi:10.4103/1673-5374.293148
50. Li H, Chen W, Gou M, et al. The relationship between TLR4/NF-κB/IL-1β signaling, cognitive impairment, and white-matter integrity in patients with stable chronic schizophrenia. *Frontiers in Psychiatry*. 2022 2022;13doi:doi:10.3389/fpsyt.2022.966657
51. Kelly JR, Minuto C, Cryan JF, Clarke G, Dinan TG. The role of the gut microbiome in the development of schizophrenia. *Schizophrenia Research*. 2021 2021;234:4-23. doi:doi:10.1016/j.schres.2020.02.010
52. Carnac T. Schizophrenia Hypothesis: Autonomic Nervous System Dysregulation of Fetal and Adult Immune Tolerance. *Front Syst Neurosci*. 2022 2022;16:844383. doi:doi:10.3389/fnsys.2022.844383
53. Langeh U, Singh S. Targeting s100b protein as a surrogate biomarker and its role in various neurological disorders. *Current Neuropharmacology*. 2021 2021;19(2):265-277. doi:doi:10.2174/1570159X18666200729100427
54. Ketharanathan T, Pereira A, Lawrence A, Everall I, Sundram S. TOLL-LIKE RECEPTOR (TLR) PATHWAY EXPRESSION IN DORSOLATERAL PREFRONTAL (BA46) AND ORBITOFRONTAL (BA11) CORTICES IN SCHIZOPHRENIA AND MOOD DISORDER. *AUSTRALIAN AND NEW ZEALAND JOURNAL OF PSYCHIATRY*. 2022-5 2022;56(1):222-222. doi:https://doi.org/10.1093/schbul/sby017.546
55. Mostafa M, Elwasify M, Fathy AA, Abdelsalam M. Toll-Like Receptor 4 Gene Polymorphisms and Susceptibility to Schizophrenia: A Case-Control Study. *Immunol Invest*. 2022-10 2022;51(7):2009-2024. doi:doi:10.1080/08820139.2022.2093118
56. Massarali A, Adhya D, Srivastava DP, Baron-Cohen S, Kotter MR. Virus-Induced Maternal Immune Activation as an Environmental Factor in the Etiology of Autism and Schizophrenia. *Frontiers in Neuroscience*. 2022 2022;16doi:doi:10.3389/fnins.2022.834058
57. Kéri S, Szabó C, Kelemen O. Antipsychotics influence Toll-like receptor (TLR) expression and its relationship with cognitive functions in schizophrenia. *Brain Behav Immun*. 2017-5 2017;62:256-264. doi:doi:10.1016/j.bbi.2016.12.011
58. Kéri S, Szabó C, Kelemen O. Uniting the neurodevelopmental and immunological hypotheses: Neuregulin 1 receptor ErbB and Toll-like receptor activation in first-episode schizophrenia. *Sci Rep*. 2017-6-23 2017;7(1):4147. doi:doi:10.1038/s41598-017-03736-3
59. Kozłowska E, Agier J, Wysokiński A, Łucka A, Sobierajska K, Brzezińska-Błaszczyk E. The expression of toll-like receptors in peripheral blood mononuclear cells is altered in schizophrenia. *Psychiatry Res*. 2019-2 2019;272:540-550. doi:doi:10.1016/j.psychres.2018.12.138
60. Li CJ. Oxidative stress and mitochondrial dysfunction in human diseases: Pathophysiology, predictive biomarkers, therapeutic. *Biomolecules*. 2020 2020;10(11):1-3. doi:doi:10.3390/biom10111558
61. Li H, Kéri S. TLR4 (Toll-like receptor-4) expression and frontal-cingulate volumes in schizophrenia. *Ideggyogy Sz*. 2020-9-30 2020;73(9):303-308. doi:doi:10.18071/isz.73.0303 %(TLR4- (Toll-like recetor-4-) expresszió és a frontocingularis régió térfogata szkizofréniában.

62. Li Z, Farias FHG, Dube U, et al. The TMEM106B FTLD-protective variant, rs1990621, is also associated with increased neuronal proportion. *Acta Neuropathologica*. 2020 2020;139(1):45-61. doi:doi:10.1007/s00401-019-02066-0
63. Liao Y, Zhang YN, Liu XL, et al. Maternal Murine Cytomegalovirus Infection during Pregnancy Up-regulates the Gene Expression of Toll-like Receptor 2 and 4 in Placenta. *CURRENT MEDICAL SCIENCE*. 2018-8 2018;38(4):632-639. doi:doi:10.1007/s11596-018-1924-z
64. Wadhawan A, Reynolds MA, Makkar H, et al. Periodontal pathogens and neuropsychiatric health. *Current Topics in Medicinal Chemistry*. 2020 2020;20(15):1353-1397. doi:doi:10.2174/1568026620666200110161105
65. Wang X, Huang J, Zhu F. Human endogenous retroviral envelope protein Syncytin-1 and inflammatory abnormalities in neuropsychological diseases. *Frontiers in Psychiatry*. 2018 2018;9doi:doi:10.3389/fpsyt.2018.00422
66. Wang X, Liu Z, Wang P, et al. Syncytin-1, an endogenous retroviral protein, triggers the activation of CRP via TLR3 signal cascade in glial cells. *Brain, Behavior, and Immunity*. 2018 2018;67:324-334. doi:doi:10.1016/j.bbi.2017.09.009
67. Chaumette B, Kebir O, Pouch J, et al. Longitudinal Analyses of Blood Transcriptome During Conversion to Psychosis. *Schizophr Bull*. 2019-1-1 2019;45(1):247-255. doi:doi:10.1093/schbul/sby009
68. Chen S, Tian L, Chen N, et al. More dampened monocytic Toll-like receptor 4 response to lipopolysaccharide and its association with cognitive function in Chinese Han first-episode patients with schizophrenia. *Schizophr Res*. 2019-4 2019;206:300-306. doi:doi:10.1016/j.schres.2018.11.001
69. Ermakov EA, Parshukova DA, Nevinsky GA, Buneva VN. Natural Catalytic IgGs Hydrolyzing Histones in Schizophrenia: Are They the Link between Humoral Immunity and Inflammation? *Int J Mol Sci*. 2020-9-30 2020;21(19)doi:doi:10.3390/ijms21197238
70. Gruchot J, Kremer D, Küry P. Neural cell responses upon exposure to human endogenous retroviruses. *Frontiers in Genetics*. 2019 2019;10doi:doi:10.3389/fgene.2019.00655
71. Herron JW, Nerurkar L, Cavanagh J. *Neuroimmune biomarkers in mental illness*. 2018. https://www.scopus.com/inward/record.uri?eid=2-s2.0-85055922300&doi=10.1007%2f7854_2018_45&partnerID=40&md5=de63c5eb267ef4c851dd484d3a39dd63
72. Huang Q, Yu F, Liao D, Xia J. Microbiota-immune system interactions in human neurological disorders. *CNS and Neurological Disorders - Drug Targets*. 2020 2020;19(7):509-526. doi:doi:10.2174/1871527319666200726222138
73. Juncal-Ruiz M, Riesco-Davila L, Vazquez-Bourgon J, et al. Expression and Functionality Study of 9 Toll-Like Receptors in 33 Drug-Naive Non-Affective First Episode Psychosis Individuals: A 3-Month Study. *INTERNATIONAL JOURNAL OF MOLECULAR SCIENCES*. 2020-9 2020;21(17)doi:doi:10.3390/ijms21176106
74. Kelly JR, Minuto C, Cryan JF, Clarke G, Dinan TG. Cross talk: The microbiota and neurodevelopmental disorders. *Frontiers in Neuroscience*. 2017 2017;11doi:doi:10.3389/fnins.2017.00490
75. Kéri S, Szabó C, Kelemen O. Erratum: Uniting the neurodevelopmental and immunological hypotheses: Neuregulin 1 receptor ErbB and Toll-like receptor activation in first-episode schizophrenia. *Sci Rep*. 2017-8-24 2017;7(1):9758. doi:doi:10.1038/s41598-017-09610-6
76. Keri S, Szabo C, Kelemen O. Uniting the neurodevelopmental and immunological hypotheses: Neuregulin 1 receptor ErbB and Toll-like receptor activation in first-episode schizophrenia (vol 7, 4141, 2017). *SCIENTIFIC REPORTS*. 2017-8-24 2017;7doi:doi:10.1038/s41598-017-09610-6
77. Ashdown H, Dumont Y, Ng M, Poole S, Boksa P, Luheshi GN. The role of cytokines in mediating effects of prenatal infection on the fetus: implications for schizophrenia. *MOLECULAR PSYCHIATRY*. 2006-1 2006;11(1):47-55. doi:doi:10.1038/sj.mp.4001748
78. Ibi D, Nagai T, Kitahara Y, et al. Neonatal polyI:C treatment in mice results in schizophrenia-like behavioral and neurochemical abnormalities in adulthood. *NEUROSCIENCE RESEARCH*. 2009-7 2009;64(3):297-305. doi:doi:10.1016/j.neures.2009.03.015
79. Chang SH, Chiang SY, Chiu CC, et al. Expression of anti-cardiolipin antibodies and inflammatory associated factors in patients with schizophrenia. *Psychiatry Res*. 2011-5-30 2011;187(3):341-6. doi:doi:10.1016/j.psychres.2010.04.049
80. McKernan DP, Dennison U, Cryan JF, Dinan TG. ALTERED TOLL-LIKE RECEPTOR RESPONSES IN SCHIZOPHRENIA AND BIPOLAR DISORDER PATIENTS. *IRISH JOURNAL OF MEDICAL SCIENCE*. 2011-2 2011;180:63-63. doi:doi:
81. McKernan DP, Dennison U, Gaszner G, Cryan JF, Dinan TG. Enhanced peripheral toll-like receptor responses in psychosis: further evidence of a pro-inflammatory phenotype. *Transl Psychiatry*. 2011-8-30 2011;1(8):e36. doi:doi:10.1038/tp.2011.37
82. Bolon B. Cellular and Molecular Mechanisms of Autoimmune Disease. *Toxicologic Pathology*. 2012 2012;40(2):216-229. doi:doi:10.1177/0192623311428481

83. Krause D, Wagner J, Matz J, et al. Monocytic HLA DR antigens in schizophrenic patients. *Neurosci Res*. 2012-1 2012;72(1):87-93. doi:doi:10.1016/j.neures.2011.09.004
84. Lee SM, Kang WS, Paik JW, Kim JW, Song JY. Toll-like receptor 2 polymorphisms are associated with poor concentration in schizophrenia patients in a Korean population. *EUROPEAN NEUROPSYCHOPHARMACOLOGY*. 2012-10 2012;22:S156-S157. doi:doi:
85. Müller N, Wagner JK, Krause D, et al. Impaired monocyte activation in schizophrenia. *Psychiatry Res*. 2012-8-15 2012;198(3):341-6. doi:doi:10.1016/j.psychres.2011.12.049
86. Aguirre A, Maturana CJ, Harcha PA, Sáez JC. Possible involvement of TLRs and hemichannels in stress-induced CNS dysfunction via mastocytes, and glia activation. *Mediators Inflamm*. 2013 2013;2013:893521. doi:doi:10.1155/2013/893521
87. Barichello T, Generoso JS, Milioli G, Elias SG, Teixeira AL. Pathophysiology of bacterial infection of the central nervous system and its putative role in the pathogenesis of behavioral changes. *Revista Brasileira de Psiquiatria*. 2013 2013;35(1):81-87. doi:doi:10.1016/j.rbp.2012.11.003
88. Chew LJ, Fusar-Poli P, Schmitz T. Oligodendroglial Alterations and the Role of Microglia in White Matter Injury: Relevance to Schizophrenia. *DEVELOPMENTAL NEUROSCIENCE*. 2013 2013;35(2):102-129. doi:doi:10.1159/000346157
89. Hopper AT, Jones KA, Campbell BM, Li G. *Neuroinflammation in mood disorders: Mechanisms and drug targets*. 2013. https://www.scopus.com/inward/record.uri?eid=2-s2.0-84884144768&doi=10.1016%2fB978-0-12-417150-3.00020-X&partnerID=40&md5=1f173f5cf1ba0164664da8a718dfd462
90. Kang WS, Park JK, Lee SM, Kim SK, Park HJ, Kim JW. Association between genetic polymorphisms of Toll-like receptor 2 (TLR2) and schizophrenia in the Korean population. *Gene*. 2013-9-10 2013;526(2):182-6. doi:doi:10.1016/j.gene.2013.04.058
91. Liu HY, Hong YF, Huang CM, Chen CY, Huang TN, Hsueh YP. TLR7 Negatively Regulates Dendrite Outgrowth through the Myd88-c-Fos-IL-6 Pathway. *JOURNAL OF NEUROSCIENCE*. 2013-7-10 2013;33(28):11479-11493. doi:doi:10.1523/JNEUROSCI.5566-12.2013
92. Lucas K, Maes M. Role of the Toll Like receptor (TLR) radical cycle in chronic inflammation: possible treatments targeting the TLR4 pathway. *Mol Neurobiol*. 2013-8 2013;48(1):190-204. doi:doi:10.1007/s12035-013-8425-7
93. Shastri A, Bonifati DM, Kishore U. Innate immunity and neuroinflammation. *Mediators of Inflammation*. 2013 2013;2013doi:doi:10.1155/2013/342931
94. Dammann O, Leviton A. Intermittent or sustained systemic inflammation and the preterm brain. *Pediatric Research*. 2014 2014;75(3):376-380. doi:doi:10.1038/pr.2013.238
95. Lin CW, Chen CY, Cheng SJ, Hu HT, Hsueh YP. Sarm1 deficiency impairs synaptic function and leads to behavioral deficits, which can be ameliorated by an mGluR allosteric modulator. *Front Cell Neurosci*. 2014 2014;8:87. doi:doi:10.3389/fncel.2014.00087
96. Nagai T. Effects of Genetic and Environmental Factors on Neuropsychological Development. *YAKUGAKU ZASSHI-JOURNAL OF THE PHARMACEUTICAL SOCIETY OF JAPAN*. 2014-10 2014;134(10):1029-1035. doi:doi:10.1248/yakushi.14-00182
97. Xia YC, Qi FF, Zou JT, Yang JH, Yao ZB. Influenza vaccination during early pregnancy contributes to neurogenesis and behavioral function in offspring. *BRAIN BEHAVIOR AND IMMUNITY*. 2014-11 2014;42:212-221. doi:doi:10.1016/j.bbi.2014.06.202
98. Yu H, Bi WJ, Liu CX, et al. Protein-interaction-network-based analysis for genome-wide association analysis of schizophrenia in Han Chinese population. *JOURNAL OF PSYCHIATRIC RESEARCH*. 2014-3 2014;50:73-78. doi:doi:10.1016/j.jpsychires.2013.11.014
99. Crisafulli C, Drago A, Calabrò M, Spina E, Serretti A. A molecular pathway analysis informs the genetic background at risk for schizophrenia. *Prog Neuropsychopharmacol Biol Psychiatry*. 2015-6-3 2015;59:21-30. doi:doi:10.1016/j.pnpbp.2014.12.009
100. de Baumont A, Maschietto M, Lima L, et al. Innate immune response is differentially dysregulated between bipolar disease and schizophrenia. *Schizophrenia Research*. 2015 2015;161(2):215-221. doi:doi:10.1016/j.schres.2014.10.055
101. Szabo A. Psychedelics and Immunomodulation: Novel Approaches and Therapeutic Opportunities. *Front Immunol*. 2015 2015;6:358. doi:doi:10.3389/fimmu.2015.00358
102. Bernstein HG, Piontkewitz Y, Keilhoff G. Commentary: Maternal immune activation evoked by polyinosinic: Polycytidylic acid does not evoke microglial cell activation in the embryo. *Frontiers in Cellular Neuroscience*. 2016 2016;10doi:doi:10.3389/fncel.2015.00301
103. Caso JR, Balanzá-Martínez V, Palomo T, García-Bueno B. The Microbiota and Gut-Brain Axis: Contributions to the Immunopathogenesis of Schizophrenia. *Curr Pharm Des*. 2016 2016;22(40):6122-6133. doi:doi:10.2174/1381612822666160906160911

104. Chrobak AA, Nowakowski J, Dudek D. Interactions between the gut microbiome and the central nervous system and their role in schizophrenia, bipolar disorder and depression. *Archives of Psychiatry and Psychotherapy*. 2016 2016;18(2):5-11. doi:doi:10.12740/APP/62962
105. García Bueno B, Caso JR, Madrigal JLM, Leza JC. Innate immune receptor Toll-like receptor 4 signalling in neuropsychiatric diseases. *Neuroscience and Biobehavioral Reviews*. 2016 2016;64:134-147. doi:doi:10.1016/j.neubiorev.2016.02.013
106. García-Bueno B, Gassó P, MacDowell KS, et al. Evidence of activation of the Toll-like receptor-4 proinflammatory pathway in patients with schizophrenia. *J Psychiatry Neurosci*. 2016-4 2016;41(3):E46-55. doi:doi:10.1503/jpn.150195
107. Lee Y, Jeon SJ, Choi JW, Ryu JH. Activation of maternal toll-like receptor-2 causes social deficits and memory impairment in female offspring. *NEUROREPORT*. 2016-3-2 2016;27(4):224-229. doi:doi:10.1097/WNR.0000000000000517
108. Orhan F, Bhat M, berg K, et al. Tryptophan Metabolism Along the Kynurenine Pathway Downstream of Toll-like Receptor Stimulation in Peripheral Monocytes. *SCANDINAVIAN JOURNAL OF IMMUNOLOGY*. 2016-11 2016;84(5):262-271. doi:doi:10.1111/sji.12479
109. Severance EG, Yolken RH, Eaton WW. Autoimmune diseases, gastrointestinal disorders and the microbiome in schizophrenia: more than a gut feeling. *SCHIZOPHRENIA RESEARCH*. 2016-9 2016;176(1):23-35. doi:doi:10.1016/j.schres.2014.06.027
110. Sharma A, Goyal R. Experimental brain ischemic preconditioning: A concept to putative targets. *CNS and Neurological Disorders - Drug Targets*. 2016 2016;15(4):489-495. doi:doi:10.2174/1871527314666150821112228
111. Adorjan I, Sun B, Feher V, et al. Evidence for Decreased Density of Calretinin-Immunopositive Neurons in the Caudate Nucleus in Patients With Schizophrenia. *Frontiers in Neuroanatomy*. 2020 2020;14doi:doi:10.3389/fnana.2020.581685
112. Alam R, Abdolmaleky HM, Zhou JR. Microbiome, inflammation, epigenetic alterations, and mental diseases. *American Journal of Medical Genetics, Part B: Neuropsychiatric Genetics*. 2017 2017;174(6):651-660. doi:doi:10.1002/ajmg.b.32567
113. Ardalan M, Chumak T, Vexler Z, Mallard C. Sex-dependent effects of perinatal inflammation on the brain: Implication for neuro-psychiatric disorders. *International Journal of Molecular Sciences*. 2019 2019;20(9)doi:doi:10.3390/ijms20092270
114. Balaji R, Subbanna M, Shivakumar V, Abdul F, Venkatasubramanian G, Debnath M. Pattern of expression of Toll like receptor (TLR)-3 and -4 genes in drug-naïve and antipsychotic treated patients diagnosed with schizophrenia. *Psychiatry Res*. 2020-3 2020;285:112727. doi:doi:10.1016/j.psychres.2019.112727
115. Chase KA, Melbourne JK, Rosen C, et al. Traumagenics: At the intersect of childhood trauma, immunity and psychosis. *Psychiatry Research*. 2019 2019;273:369-377. doi:doi:10.1016/j.psychres.2018.12.097
116. Zagorska A, Marcinkowska M, Jamrozik M, Wisniowska B, Pasko P. From probiotics to psychobiotics - the gut-brain axis in psychiatric disorders. *BENEFICIAL MICROBES*. 2020 2020;11(8):717-732. doi:doi:10.3920/BM2020.0063
117. Lashgari NA, Roudsari NM, Shamsnia HS, Shayan M, Momtaz S, Abdolghaffari AH. TLR/mTOR inflammatory signaling pathway: novel insight for the treatment of schizophrenia. *Can J Physiol Pharmacol*. Mar 1 2024;102(3):150-160. doi:10.1139/cjpp-2023-0107
118. Chaudhary A, Mehra P, Keshri AK, Rawat SS, Mishra A, Prasad A. The Emerging Role of Toll-Like Receptor-Mediated Neuroinflammatory Signals in Psychiatric Disorders and Acquired Epilepsy. *Mol Neurobiol*. Mar 2024;61(3):1527-1542. doi:10.1007/s12035-023-03639-7
119. Ayari F, Chaaben AB, Abaza H, et al. Association between genetic variants of TLR2, TLR4, TLR9 and schizophrenia. *Encephale*. Apr 2024;50(2):178-184. doi:10.1016/j.encep.2023.05.004
120. Tseng CC, Wang SC, Yang YC, et al. Aberrant Histone Modification of TNFAIP3, TLR4, TNIP2, miR-146a, and miR-155 in Major Depressive Disorder. *Mol Neurobiol*. Aug 2023;60(8):4753-4760. doi:10.1007/s12035-023-03374-z
121. Sotelo-Ramírez CE, Camarena B, Sanabrais-Jiménez MA, et al. Toll-Like Receptor (TLR) 1, 2, and 6 Gene Polymorphisms Support Evidence of Innate Immune Factors in Schizophrenia. *Neuropsychiatr Dis Treat*. 2023;19:2353-2361. doi:10.2147/ndt.S420952
122. Tsai SY, Sajatovic M, Chen PH, Huang YJ, Chung KH. Increased proportions of circulating pro-inflammatory monocytes and macrophages expressing toll-like receptor 4 in individuals with schizophrenia and bipolar disorder receiving medication. *Psychiatry Clin Neurosci*. Dec 2023;77(12):672-673. doi:10.1111/pcn.13599
123. Patlola SR, Donohoe G, McKernan DP. Counting the Toll of Inflammation on Schizophrenia-A Potential Role for Toll-like Receptors. *Biomolecules*. Jul 30 2023;13(8)doi:10.3390/biom13081188
124. Chung IH, Huang YS, Fang TH, Chen CH. Whole Genome Sequencing Revealed Inherited Rare Oligogenic Variants Contributing to Schizophrenia and Major Depressive Disorder in Two Families. *Int J Mol Sci*. Jul 21 2023;24(14)doi:10.3390/ijms241411777
