# Supplementary material for: Monocytic TLR4 expression and activation in schizophrenia: A systematic review and meta-analysis
Source: PLoS One. 2025 Mar 28;20(3):e0319171. doi: 10.1371/journal.pone.0319171 (PMC11952227; doi:10.1371/journal.pone.0319171)
Supplement: Attachment — Submitted filename: PONE-D-24-33258.docx [file pone.0319171.s004.docx]

**Manuscript number: PONE-D-24-33258**

**Manuscript title: Monocytic TLR4 expression and activation in schizophrenia: a systematic review and meta-analysis**

This is an interesting topic and an area to expand further research. While I believe the work presents an interesting topic, there are multiple issues with the content of the paper.

- Search strategy
- Please set up the method and describe how to select references and establish the logic or focus of this review. It must be comprehensive or logical in the focused topic as much as you can.
- Consider adding a summarizing table(s) and/or figures presenting some of the discussed mechanism and the flowchart of the study selection process.
