# Supplementary material for: Monocytic TLR4 expression and activation in schizophrenia: A systematic review and meta-analysis
Source: PLoS One. 2025 Mar 28;20(3):e0319171. doi: 10.1371/journal.pone.0319171 (PMC11952227; doi:10.1371/journal.pone.0319171)
Supplement: Attachment — Submitted filename: Response to Reviewers.docx [file pone.0319171.s005.docx]

**PONE-D-24-33258**

**Monocytic TLR4 expression and activation in schizophrenia: a systematic review and meta-analysis**

**PLOS ONE**

**Dear Dr. Akhondzadeh,**

**Thank you for submitting your manuscript to PLOS ONE. After careful consideration, we feel that it has merit but does not fully meet PLOS ONE’s publication criteria as it currently stands. Therefore, we invite you to submit a revised version of the manuscript that addresses the points raised during the review process.**

**Please include the following items when submitting your revised manuscript:**

**We look forward to receiving your revised manuscript.**

**Kind regards,**

**Ahmad Salimi**

**Academic Editor**

**PLOS ONE**

- **Reply**: Dear Dr. Ahmad Salimi,

Thank you very much for providing us with the opportunity to strengthen our research. We sincerely appreciate all the precious comments from you and the respected reviewers. Having carefully considered the comments and suggestions, we have made all the relevant changes to our manuscript as outlined below in an itemized, point-by-point manner. We sincerely hope that these changes meet the approval criteria of the esteemed reviewers and the editorial board.

Best Regards,

- ***Shahin Akhondzadeh****; Pharm. D., PhD*

*Full Professor of Neuroscience; Psychiatry and Psychology Research Center, Roozbeh Hospital, Tehran University of Medical Sciences, Tehran, Iran;* [sakhond@yahoo.com](mailto:sakhond@yahoo.com)

- ***Seyed Behnamedin Jameie****; PhD*

*Full Professor of Anatomy and Neuroscience; Neuroscience Research Center and Department of Anatomy, Iran University of Medical Sciences, Tehran, Iran;* [jameie.sb@iums.ac.ir](mailto:jameie.sb@iums.ac.ir); [behjame@gmail.com](mailto:behjame@gmail.com)

# **Journal Requirements:**

1. **When submitting your revision, we need you to address these additional requirements. Please ensure that your manuscript meets PLOS ONE's style requirements, including those for file naming. The PLOS ONE style templates can be found at** [**https://journals.plos.org/plosone/s/file?id=wjVg/PLOSOne_formatting_sample_main_body.pdf**](https://journals.plos.org/plosone/s/file?id=wjVg/PLOSOne_formatting_sample_main_body.pdf) **and**[**https://journals.plos.org/plosone/s/file?id=ba62/PLOSOne_formatting_sample_title_authors_affiliations.pdf**](https://journals.plos.org/plosone/s/file?id=ba62/PLOSOne_formatting_sample_title_authors_affiliations.pdf)
   - **Reply**: Thank you for your time and effort. The manuscript meets PLOS ONE's style requirements.
2. **In the online submission form, you indicated that The data underlying the results presented in the study are available from the corresponding author. Contact information:** [**sakhond@yahoo.com**](mailto:sakhond@yahoo.com)**. All PLOS journals now require all data underlying the findings described in their manuscript to be freely available to other researchers, either a. In a public repository, b. Within the manuscript itself, or c. Uploaded as supplementary information. This policy applies to all data except where public deposition would breach compliance with the protocol approved by your research ethics board. If your data cannot be made publicly available for ethical or legal reasons (e.g., public availability would compromise patient privacy), please explain your reasons on resubmission and your exemption request will be escalated for approval.**
   - **Reply**: Thank you for mentioning this issue. In the revised manuscript, the data underlying the findings is uploaded as *supplementary information*. Accordingly, we revised the Data Availability Statement.
3. **PLOS requires an ORCID iD for the corresponding author in Editorial Manager on papers submitted after December 6th, 2016. Please ensure that you have an ORCID iD and that it is validated in Editorial Manager. To do this, go to ‘Update my Information’ (in the upper left-hand corner of the main menu), and click on the Fetch/Validate link next to the ORCID field. This will take you to the ORCID site and allow you to create a new iD or authenticate a pre-existing iD in Editorial Manager.**
   - **Reply**: Thank you very much. We will make sure that the ORDID IDs for corresponding authors are provided in Editorial Manager. However, here, we provide their ORCIDs.
     - Melika Jameie (First author): [0000-0002-2028-9935](https://orcid.org/0000-0002-2028-9935)
     - Seyed Behnamedin Jameie (Corresponding author): [0000-0003-2062-4155](https://orcid.org/0000-0003-2062-4155)
     - Shahin Akhondzadeh (Corresponding author): [0000-0002-2277-5101](https://orcid.org/0000-0002-2277-5101)
4. **Your ethics statement should only appear in the Methods section of your manuscript. If your ethics statement is written in any section besides the Methods, please delete it from any other section.**
   - **Reply**: We removed the Ethics approval and consent to participate section at the end of the manuscript. In the revised version, the ethics statement only appears in the Methods section.
5. **As required by our policy on Data Availability, please ensure your manuscript or supplementary information includes the following:**

- **A** **numbered table of all studies identified in the literature search, including those that were excluded from the analyses. For every excluded study, the table should list the reason(s) for exclusion.**
  - **Reply**: Thank you very much for your constructive feedback. According to the journal’s policy on data availability, we added this information in a new supplemental table (**Supplementary Table S6)**. This is mentioned in the text: “*Table S6 presents a numbered list of the 125* *studies identified in the literature search that proceeded to full-text screening, along with the reasons for exclusion for each excluded study*.” It should be noted that there might be more than one reason for exclusion for each excluded study.
- **If any of the included studies are unpublished, include a link (URL) to the primary source or detailed information about how the content can be accessed.**
  - **Reply**: There are no included studies that are unpublished.
- **A table of all data extracted from the primary research sources for the systematic review and/or meta-analysis. The table must include the following information for each study: Name of data extractors and date of data extraction, and Confirmation that the study was eligible to be included in the review.**
  - **Reply**: The Name of the data extractors and date of data extraction were added for the included studies in **Supplementary Table S6.** The confirmation that the study was eligible to be included is reported in **Supplementary Table S6**.
- **All data extracted from each study for the reported systematic review and/or meta-analysis that would be needed to replicate your analyses.**
  - **Reply**: All data extracted from each study for the reported systematic review and meta-analysis that would be needed to replicate our analyses can be found in Tables 1 and 2 of the manuscript. Additionally, we provided the data underlying the meta-analysis results as Supplementary information.
- **If data or supporting information were obtained from another source (e.g. correspondence with the author of the original research article), please provide the source of data and dates on which the data/information were obtained by your research group.**
  - **Reply:** All data is obtained from the main original articles. No other source has been used.
- **If applicable for your analysis, a table showing the completed risk of bias and quality/certainty assessments for each study or outcome. Please ensure this is provided for each domain or parameter assessed. For example, if you used the Cochrane risk-of-bias tool for randomized trials, provide answers to each of the signalling questions for each study. If you used GRADE to assess certainty of evidence, provide judgements about each of the quality of evidence factor. This should be provided for each outcome.**
  - **Reply**: **Table S7** provides detail information regarding the Risk of bias evaluation for the outcome of interest for each study, using the Joanna Briggs Institute (JBI) Critical Appraisal tool.
- **An explanation of how missing data were handled. This information can be included in the main text, supplementary information, or relevant data repository. Please note that providing these underlying data is a requirement for publication in this journal, and if these data are not provided your manuscript might be rejected.**
  - **Reply:** All information required for the meta-analyses was extracted from the main text or the main figures of the original manuscripts. For studies where the numbers were not reported in the main text, we included the following note in the table legends: “*As the study results were shown in the figures (not tables), the numbers were extracted from the figures using* [*https://automeris.io/WebPlotDigitizer*](https://automeris.io/WebPlotDigitizer)*/. Therefore, the extracted numbers might be slightly different from the exact numbers found by the authors*.” Notably, the underlying data is provided as supplementary information in the revised version of our manuscript.

6. **Please review your reference list to ensure that it is complete and correct. If you have cited papers that have been retracted, please include the rationale for doing so in the manuscript text, or remove these references and replace them with relevant current references. Any changes to the reference list should be mentioned in the rebuttal letter that accompanies your revised manuscript. If you need to cite a retracted article, indicate the article’s retracted status in the References list and also include a citation and full reference for the retraction notice.**
   - **Reply:** Thank you again for all your time and effort. The reference list is complete and correct.

# **Reviewer 1:**

**This is an interesting topic and an area to expand further research. While I believe the work presents an interesting topic, there are multiple issues with the content of the paper.**

1. **Search strategy**
2. **Please set up the method and describe how to select references and establish the logic or focus of this review. It must be comprehensive or logical in the focused topic as much as you can.**
3. **Consider adding a summarizing table(s) and/or figures presenting some of the discussed mechanism and the flowchart of the study selection process**.
- **Reply**: Dear reviewer,

Thank you very much for your kind words and for providing us with the opportunity to strengthen our research. We sincerely appreciate your precious comments. Having carefully considered the comments and suggestions, we have made all the relevant changes to our manuscript as outlined below in an itemized, point-by-point manner. The revisions requested have been track-changed and highlighted within the manuscript in response.

1. **Reply**: Thank you very much for your precious comment. Our main research question was to investigate the basal expression and activation pattern of TLR4 in PBMCs, including monocytes, among individuals with schizophrenia compared to HCs. We conducted a systematic search in three databases, including MEDLINE (via PubMed), Scopus, and Web of Science. Then, we used the “Patient, Intervention, Comparison, and Outcome” (PICO) model to formulate our research question [1]:

- Patient: Persons with schizophrenia
- Intervention/exposure: not applicable
- Comparison: HCs
- Outcome(s):

1. TLR4 basal expression patterns in PBMCs

2. TLR4 activation patterns in PBMCs

Then, two PICO elements were used to convert our research question into a search strategy:

a. Patient-related MeSH Terms and text word terms: (“schizophrenia”, “schizophrenic”, “schizophrenias”, “psychosis”, and “psychotic”) AND
b. Outcome-related MeSH terms and text word terms: “toll-like”, “toll-like receptor”, “toll-like receptor 4”, and “TLR”

All the information is written in the “Methods section > Objectives and review questions/ Study design, information sources, and search strategy”. However, since we taught that providing the whole search strategy within the manuscript text might be space-consuming, we provided the detailed search strategy in **Supplemental Tables S3-S5**. Sincerely, please let us know if any other clarifications are required considering the search strategy.

| **MEDLINE (via PubMed)** | No. |
| --- | --- |
| 1. (Toll-like receptors [MeSH Terms] OR “toll-like receptors” [Title/Abstract] OR TLR [Title/Abstract] OR “toll-like receptor” [Title/Abstract] OR “Toll-like” [Title/Abstract] OR “Toll-Like receptor 4” [Title/Abstract] OR TLR4 [Title/Abstract] OR TLR-4 [Title/Abstract] OR “Toll-Like receptor 4” [MeSH Terms]) | **73579** |
| 2. (Schizophrenia [MeSH Terms] OR schizophrenia [Title/Abstract] OR psychotic [Title/Abstract] OR psychosis [Title/Abstract] OR psychotic disorders [MeSH Terms] OR “psychotic disorders” [Title/Abstract] OR “psychotic disorder” [Title/Abstract] OR schizophrenias [Title/Abstract] OR schizophrenic [Title/Abstract]) | **228052** |
| #1 AND #2 | **112** |
| **Web of Science** |  |
| 1. (TS= (“Toll-like receptors”) OR TS= (“toll-like receptor”) OR TS= (TLR) OR TS= (“Toll-like”) OR TS= (“Toll-Like receptor 4”) OR TS= (TLR4) OR TS= (TLR-4)) | **87616** |
| 2. (TS= (Schizophrenia) OR TS= (psychotic) OR TS= (psychosis) OR TS= (“psychotic disorders”) OR TS= (“psychotic disorder”) OR TS= (schizophrenias) OR TS= (schizophrenic)) | **266798** |
| #1 AND #2 | **171** |
| **Scopus** |  |
| 1. (INDEXTERMS("Toll-like receptors") OR TITLE-ABS("toll-like receptors") OR TITLE-ABS(TLR) OR TITLE-ABS("toll-like receptor") OR TITLE-ABS(Toll-like) OR TITLE-ABS("Toll-Like receptor 4") OR TITLE-ABS(TLR4) OR TITLE-ABS(TLR-4) OR INDEXTERMS("Toll-Like receptor 4")) | **111233** |
| 2. (TITLE-ABS-KEY (Schizophrenia ) OR TITLE-ABS-KEY (psychotic ) OR TITLE-ABS-KEY (psychosis ) OR TITLE-ABS-KEY ("psychotic disorders" ) OR TITLE-ABS-KEY ("psychotic disorder" ) OR TITLE-ABS-KEY (schizophrenias ) OR TITLE-ABS-KEY (schizophrenic )) | **340059** |
| #1 AND #2 | **339** |

2. **Reply**: Thank you very much for your thoughtful feedback. The references were selected according to the instructions below.

- First, a total of 622 records from the three databases were initially imported into EndNote software (version X9, Clarivate Plc). Then, duplicates were removed, yielding 452 records for initial screening. Then, two independent researchers (R.S. and S.B.) screened all the 452 records using titles and abstracts. Among these articles, 125 potentially eligible articles remained for full-text screening by three independent authors (M.J., S.B., and R.S.), including one expert author (M.J.). Notably, the references of these articles were also manually searched for any potentially related article that was not retrieved initially by database searching (Hand searching method). The records that did not align with the predefined eligibility criteria were excluded. In cases of discrepancies, conflicts were resolved by reaching a consensus among all authors (including the two corresponding authors).
- Among these 125 articles, 114 did not align with the predefined eligibility criteria, leaving 11 original articles aligning with the research question. The reason for the exclusion of each study is reported in the revised version of the manuscript as **Supplementary Table S6**. The information regarding the selection of studies is reported in the “Methods section > Selection process”, as well as the “Results section > Literature search and study selection”. According to your valuable comment, to ensure integrity, completeness, and logic behind selecting the references, the table below also shows the reason(s) for excluding each single study. Please let us know of there are any other clarifications required concerning selecting the studies.

**Table S6. Studies identified in the literature search that proceeded to full-text screening**

| **Title** | **First author** | **Eligibility** | **Exclusion reason/ Extractors^[1]^** |
| --- | --- | --- | --- |
| 1. JNK signaling mediates aspects of maternal immune activation: importance of maternal genotype in relation to schizophrenia risk | R.L Openshow [2] | Exclude | Animal study |
| 2. CNS Macrophages and Infant Infections | A. Oschwald [3] | Exclude | Review |
| 3. Role of inflammation in epilepsy and neurobehavioral comorbidities: Implication for therapy | Y.N. Paudel [4] | Exclude | Review |
| 4. The role of the gut microbiota in the pathophysiology of mental and neurological disorders | M.M. Pusceddu [5] | Exclude | Review |
| 5. N-3 polyunsaturated fatty acids and clozapine abrogates poly I: C-induced immune alterations in primary hippocampal neurons | B.M.M. Ribeiro [6] | Exclude | In-vitro |
| 6. Lack of Helios During Neural Development Induces Adult Schizophrenia-Like Behaviors Associated With Aberrant Levels of the TRIF-Recruiter Protein WDFY1 | A. Sancho-Balsells [7] | Exclude | Animal study |
| 7. Targeting the NLRP3 Inflammasome-Related Pathways via Tianeptine Treatment-Suppressed Microglia Polarization to the M1 Phenotype in Lipopolysaccharide-Stimulated Cultures | J. Slusarczyk [8] | Exclude | In-vitro |
| 8. Importance of the immune system in mediating plasticity of the brain and behavior | L. Tian [9] | Exclude | Review |
| 9. Evidence of microglial activation following exposure to serum from first-onset drug-naïve schizophrenia patients | G.F.V. Rees [10] | Exclude | levels of TLR were not discussed. |
| 10. CD157 and Brain Immune System in (Patho)physiological Conditions: Focus on Brain Plasticity | O.L. Lopatina [11] | Exclude | Review |
| 11. Neuroinflammation in the dorsolateral prefrontal cortex in elderly chronic schizophrenia | I. Lopez-Gonzalez [12] | Exclude | Post-mortem study |
| 12. The Role of Immune Factors in Shaping Fetal Neurodevelopment | A. Lu-Culligan [13] | Exclude | Review |
| 13. Differential regulation of the TLR4 signaling pathway in post-mortem prefrontal cortex and cerebellum in chronic schizophrenia: Relationship with SP transcription factors | K.S. MacDowell [14] | Exclude | Post-mortem study |
| 14. Immunomodulatory effects of antipsychotic treatment on gene expression in first-episode psychosis | O. Mantere [15] | Exclude | Discussed psychosis, not schizophrenia specifically |
| 15. Emerging role of innate B1 cells in the pathophysiology of autoimmune and neuroimmune diseases: Association with inflammation, oxidative and nitrosative stress and autoimmune responses | G. Morris [16] | Exclude | Review |
| 16. Active psychosis and pro-inflammatory cytokines in first episode of psychosis | G. Pardo-de-Santayana [17] | Exclude | TLR was not discussed, cytokines were investigated |
| 17. Anxiety-like behavior and microglial activation in the amygdala after acute neuroinflammation induced by microbial neuraminidase | A. Leon-Rodriguez [18] | Exclude | Animal study |
| 18. Association between TLR2 polymorphisms (- 196-174 Ins/Del, R677W, R753Q, and P631H) and schizophrenia in a Tunisian population | Y. Aflouk [19] | Exclude | Levels of TLR were not discussed, TLR2 polymorphisms were only discussed |
| 19. Association of complement component 4 with neuroimmune abnormalities in the subventricular zone in schizophrenia and autism spectrum disorders | T. C. M. Mou [20] | Exclude | No TLR was discussed, and complement component 4 was explained |
| 20. The association of inflammatory status and immunological parameters with single-nucleotide polymorphisms of cytokine and Toll-like receptor genes in patients with schizophrenia | I. K. Malashenkova [21] | Exclude | No levels of TLR were discussed, TLR polymorphisms were only discussed |
| 21. Association of TLR-4 896A/G, TLR-4 1196C/T, and TLR-9 C/T polymorphism with schizophrenia in Indian Bengalee patient | J. Gurung [22] | Exclude | No levels of TLR were discussed, TLR polymorphisms were only discussed |
| 22. Association of toll-like receptor 2 gene polymorphism (rs3804099) with susceptibility to Schizophrenia risk in the Dogra population of Jammu region, North India | I. Sharma [23] | Exclude | No levels of TLR were discussed, TLR polymorphisms were only discussed |
| 23. Astrocyte Bioenergetics and Major Psychiatric Disorders | I. V. Maly [24] | Exclude | Book Series |
| 24. Charting the proteome landscape in major psychiatric disorders: From biomarkers to biological pathways towards drug discovery | Fern, B. S. es [25] | Exclude | systematic review and comprehensive *in silico* analysis |
| 25. Cognitive impairment in psychiatric diseases: Biomarkers of diagnosis, treatment, and prevention | Y. Wang [26] | Exclude | Review |
| 26. Consequences of Viral Infection and Cytokine Production During Pregnancy on Brain Development in Offspring | D. Elgueta [27] | Exclude | Review |
| 27. Differential Effects of Toll-Like Receptor Activation and Differential Mediation by MAP Kinases of Immune Responses in Microglial Cells | J. Kwon, C. Arsenis [28] | Exclude | In-vitro |
| 28. Dysfunctional monocytic toll-like receptor 4 signaling pathway and cognitive deficits in chronic schizophrenia patients with tardive dyskinesia | N. Li [29] | Include | * Independent data extraction (February 2024): M.J., R.S., S.B.  * Conflicts: M.A. and M.J. |
| 29. Early life Adversity, functional connectivity and cognitive performance in Schizophrenia: The mediating role of IL-6 | S. King [30] | Exclude | No levels of TLR were discussed |
| 30. Editorial: Cognitive Dysfunctions in Psychiatric Disorders: Brain-Immune Interaction Mechanisms and Integrative Therapeutic Approaches | H. Xu [31] | Exclude | Editorial |
| 31. Editorial: The inflammation markers in schizophrenia and bipolar disorder: Do we have promising results? | A. Arslan [32] | Exclude | Editorial |
| 32. Environmental Risk Factors for Schizophrenia and Bipolar Disorder and Their Relationship to Genetic Risk: Current Knowledge and Future Directions | N. Robinson [33] | Exclude | Review |
| 33. First Episode Psychosis and Schizophrenia Are Systemic Neuro-Immune Disorders Triggered by a Biotic Stimulus in Individuals with Reduced Immune Regulation and Neuroprotection | M. Maes [34] | Exclude | Secondary analysis, no data on TLR |
| 34. Gut microbiota in psychiatric disorders: Better understanding or more complexity to be resolved? | B. Misiak [35] | Exclude | Editorial |
| 35. Human endogenous retrovirus W family envelope protein (HERV-W env) facilitates the production of TNF-α and IL-10 by inhibiting MyD88s in glial cells | X. Wang [36] | Exclude | In-vitro |
| 36. Identifying crucial biomarkers in peripheral blood of schizophrenia and screening therapeutic agents by comprehensive bioinformatics analysis | M. Xie [37] | Exclude | Secondary analysis, no distinct data on the levels of TLR |
| 37. Immune System Abnormalities in Schizophrenia: An Integrative View and Translational Perspectives | E. Ermakov [38] | Exclude | Review |
| 38. Impact of SARS-CoV-2 on Host Factors Involved in Mental Disorders | R. Rhoades [39] | Exclude | Review |
| 39. Influenza Virus Infection during Pregnancy as a Trigger of Acute and Chronic Complications | O. Oseghale [40] | Exclude | Review |
| 40. Involvement of TCF7L2 in generation of morphine-induced antinociceptive tolerance and hyperalgesia by modulating TLR4/ NF-κB/NLRP3 in microglia | J. Chen [41] | Exclude | Animal study |
| 41. A loss of mature microglial markers without immune activation in schizophrenia | G. J. L. J. Snijders [42] | Exclude | Review |
| 42. Maternal inflammation and its ramifications on fetal neurodevelopment | H.K. Kwon [43] | Exclude | Review |
| 43. Modulatory Effect of Gut Microbiota on the Gut-Brain, Gut-Bone Axes, and the Impact of Cannabinoids | I. Ibrahim [44] | Exclude | Review |
| 44. Negative Regulation of the IL-1 System by IL-1R2 and IL-1R8: Relevance in Pathophysiology and Disease | D. Supino [45] | Exclude | Review |
| 45. Neurobiological Highlights of Cognitive Impairment in Psychiatric Disorders | A. Morozova [46] | Exclude | Review |
| 46. Peripheral NF-κB dysregulation in people with schizophrenia drives inflammation: putative anti-inflammatory functions of NF-κB kinases | C.E. Murphy [47] | Exclude | Evaluating TLR4 gene expression in peripheral blood leukocytes (not inclusively in PBMC) |
| 47. A Possible Causal Involvement of Neuroinflammatory, Purinergic P2X7 Receptors in Psychiatric Disorders | Y. Zhang [48] | Exclude | Review |
| 48. Potential Neurocognitive Symptoms Due to Respiratory Syncytial Virus Infection | C.A. Andrade [49] | Exclude | Review |
| 49. Psychosis symptoms following aberrant immunity in the brain | A. Ozaki [50] | Exclude | Editorial |
| 50. The relationship between TLR4/NF-κB/IL-1β signaling, cognitive impairment, and white-matter integrity in patients with stable chronic schizophrenia | H. Li [51] | Included | * Independent data extraction (February 2024): M.J., R.S., S.B.  * Conflicts: M.A. and M.J. |
| 51. The role of the gut microbiome in the development of schizophrenia | J.R. Kelly [52] | Exclude | Review |
| 52. Schizophrenia Hypothesis: Autonomic Nervous System Dysregulation of Fetal and Adult Immune Tolerance | T. Carnac [53] | Exclude | Review |
| 53. Targeting s100b protein as a surrogate biomarker and its role in various neurological disorders | U. Langeh [54] | Exclude | Review |
| 54. TOLL-LIKE RECEPTOR (TLR) PATHWAY EXPRESSION IN DORSOLATERAL PREFRONTAL (BA46) AND ORBITOFRONTAL (BA11) CORTICES IN SCHIZOPHRENIA AND MOOD DISORDER | T. Ketharanathan [55] | Exclude | Post-mortem study on schizophrenia |
| 55. Toll-Like Receptor 4 Gene Polymorphisms and Susceptibility to Schizophrenia: A Case-Control Study | M. Mostafa [56] | Exclude | No levels of TLR were discussed, TLR polymorphisms were only discussed |
| 56. Virus-Induced Maternal Immune Activation as an Environmental Factor in the Etiology of Autism and Schizophrenia | A. Massrali [57] | Exclude | Review |
| 57. Antipsychotics influence Toll-like receptor (TLR) expression and its relationship with cognitive functions in schizophrenia | S. Keri [58] | Include | * Independent data extraction (February 2024): M.J., R.S., S.B.  * Conflicts: M.A. and M.J. |
| 58. Uniting the neurodevelopmental and immunological hypotheses: Neuregulin 1 receptor ErbB and Toll-like receptor activation in first-episode schizophrenia | S. Keri [59] | Exclude | Erratum |
| 59. The expression of toll-like receptors in peripheral blood mononuclear cells is altered in schizophrenia | E. Kozlowska [60] | Include | * Independent data extraction (February 2024): M.J., R.S., S.B.  * Conflicts: M.A. and M.J. |
| 60. Oxidative stress and mitochondrial dysfunction in human diseases: Pathophysiology, predictive biomarkers, therapeutic | C.J. Li [61] | Exclude | Editorial |
| 61. TLR4 (Toll-like receptor-4) expression and frontal-cingulate volumes in schizophrenia | H. Li [62] | Exclude | Non-English |
| 62. The TMEM106B FTLD-protective variant, rs1990621, is also associated with increased neuronal proportion | Z. Li [63] | Exclude | No TLRs were measured |
| 63. Maternal Murine Cytomegalovirus Infection during Pregnancy Up-regulates the Gene Expression of Toll-like Receptor 2 and 4 in Placenta | Y. Liao [64] | Exclude | Animal study |
| 64. Periodontal pathogens and neuropsychiatric health | A. Wadhawan [65] | Exclude | Review |
| 65. Human endogenous retroviral envelope protein Syncytin-1 and inflammatory abnormalities in neuropsychological diseases | X. Wang [66] | Exclude | Review |
| 66. Syncytin-1, an endogenous retroviral protein, triggers the activation of CRP via TLR3 signal cascade in glial cells | X. Wang [67] | Exclude | In-vitro |
| 67. Longitudinal Analyses of Blood Transcriptome During Conversion to Psychosis | B. Chaumette [68] | Exclude | No TLRs were measured |
| 68. More dampened monocytic Toll-like receptor 4 response to lipopolysaccharide and its association with cognitive function in Chinese Han first-episode patients with schizophrenia | S. Chen [69] | Include | * Independent data extraction (February 2024): M.J., R.S., S.B.  * Conflicts: M.A. and M.J. |
| 69. Natural Catalytic IgGs Hydrolyzing Histones in Schizophrenia: Are They the Link between Humoral Immunity and Inflammation? | E.A. Ermakov [70] | Exclude | No TLRs were measured |
| 70. Neural cell responses upon exposure to human endogenous retroviruses | J. Gruchot [71] | Exclude | Review |
| 71. Neuroimmune biomarkers in mental illness | J.W. Herron [72] | Exclude | Review |
| 72. Microbiota-immune system interactions in human neurological disorders | Q. Huang [73] | Exclude | Review |
| 73. Expression and Functionality Study of 9 Toll-Like Receptors in 33 Drug-Naive Non-Affective First Episode Psychosis Individuals: A 3-Month Study | M. Juncal-Ruiz [74] | Exclude | Discussed psychosis, not schizophrenia specifically |
| 74. Cross talk: The microbiota and neurodevelopmental disorders | J.R. Kelly [75] | Exclude | Review |
| 75. Erratum: Uniting the neurodevelopmental and immunological hypotheses: Neuregulin 1 receptor ErbB and Toll-like receptor activation in first-episode schizophrenia | S. Keri [76] | Exclude | Erratum |
| 76. Uniting the neurodevelopmental and immunological hypotheses: Neuregulin 1 receptor ErbB and Toll-like receptor activation in first-episode schizophrenia | S. Keri [77] | Include | * Independent data extraction (February 2024): M.J., R.S., S.B.  * Conflicts: M.A. and M.J. |
| 77. The role of cytokines in mediating effects of prenatal infection on the fetus: implications for schizophrenia | H. Ashdown [78] | Exclude | Review |
| 78. Neonatal polyI:C treatment in mice results in schizophrenia-like behavioral and neurochemical abnormalities in adulthood | D. Ibi [79] | Exclude | Animal study |
| 79. Expression of anti-cardiolipin antibodies and inflammatory associated factors in patients with schizophrenia | SH. Chang [80] | Include | * Independent data extraction (February 2024): M.J., R.S., S.B.  * Conflicts: M.A. and M.J. |
| 80. ALTERED TOLL-LIKE RECEPTOR RESPONSES IN SCHIZOPHRENIA AND BIPOLAR DISORDER PATIENTS | D.P. McKernan [81] | Exclude | Review |
| 81. Enhanced peripheral toll-like receptor responses in psychosis: further evidence of a pro-inflammatory phenotype | D. P. McKernan [82] | Exclude | TLR-4 was not measured |
| 82. Cellular and Molecular Mechanisms of Autoimmune Disease | B. Bolon [83] | Exclude | Review |
| 83. Monocytic HLA DR antigens in schizophrenic patients | D. Krause [84] | Exclude | TLR-4 was not measured |
| 84. Toll-like receptor 2 polymorphisms are associated with poor concentration in schizophrenia patients in a Korean population | S. M. Lee [85] | Exclude | TLR-4 was not measured |
| 85. Impaired monocyte activation in schizophrenia | N. Müller [86] | Include | * Independent data extraction (February 2024): M.J., R.S., S.B.  * Conflicts: M.A. and M.J. |
| 86. Possible involvement of TLRs and hemichannels in stress-induced CNS dysfunction via mastocytes, and glia activation | A. Aguirre [87] | Exclude | Review |
| 87. Pathophysiology of bacterial infection of the central nervous system and its putative role in the pathogenesis of behavioral changes | T. Barichello [88] | Exclude | Review |
| 88. Oligodendroglial Alterations and the Role of Microglia in White Matter Injury: Relevance to Schizophrenia | L. J. Chew [89] | Exclude | Review |
| 89. Neuroinflammation in mood disorders: Mechanisms and drug targets | A. T. Hopper [90] | Exclude | Review |
| 90. Association between genetic polymorphisms of Toll-like receptor 2 (TLR2) and schizophrenia in the Korean population | W. S. Kang [91] | Exclude | TLR-4 was not measured |
| 91. TLR7 Negatively Regulates Dendrite Outgrowth through the Myd88-c-Fos-IL-6 Pathway | H. Y. Liu [92] | Exclude | Animal study and in-vitro |
| 92. Role of the Toll Like receptor (TLR) radical cycle in chronic inflammation: possible treatments targeting the TLR4 pathway | K. Lucas [93] | Exclude | Review |
| 93. Innate immunity and neuroinflammation | A. Shastri [94] | Exclude | Review |
| 94. Intermittent or sustained systemic inflammation and the preterm brain | O. Dammann [95] | Exclude | Review |
| 95. Sarm1 deficiency impairs synaptic function and leads to behavioral deficits, which can be ameliorated by an mGluR allosteric modulator | C. W. Lin [96] | Exclude | No TLR were measured |
| 96. Effects of Genetic and Environmental Factors on Neuropsychological Development | T. Nagai [97] | Exclude | Review |
| 97. Influenza vaccination during early pregnancy contributes to neurogenesis and behavioral function in offspring | Y. C. Xia [98] | Exclude | Animal study |
| 98. Protein-interaction-network-based analysis for genome-wide association analysis of schizophrenia in Han Chinese population | H. Yu [99] | Exclude | No TLR were measured |
| 99. A molecular pathway analysis informs the genetic background at risk for schizophrenia | C. Crisafulli [100] | Exclude | This article discussed molecular pathways and no TLR were measured. |
| 100. Innate immune response is differentially dysregulated between bipolar disease and schizophrenia | A. de Baumont [101] | Exclude | No TLR were measured |
| 101. Psychedelics and Immunomodulation: Novel Approaches and Therapeutic Opportunities | A. Szabo [102] | Exclude | Review |
| 102. Commentary: Maternal immune activation evoked by polyinosinic: Polycytidylic acid does not evoke microglial cell activation in the embryo | H. G. Bernstein [103] | Exclude | Commentary |
| 103. The Microbiota and Gut-Brain Axis: Contributions to the Immunopathogenesis of Schizophrenia | J. R. Caso [104] | Exclude | Review |
| 104. Interactions between the gut microbiome and the central nervous system and their role in schizophrenia, bipolar disorder and depression | A. A. Chrobak [105] | Exclude | Review |
| 105. Innate immune receptor Toll-like receptor 4 signaling in neuropsychiatric diseases | B. García Bueno [106] | Exclude | Review |
| 106. Evidence of activation of the Toll-like receptor-4 proinflammatory pathway in patients with schizophrenia | B. García-Bueno [107] | Exclude | Post-mortem study |
| 107. Activation of maternal toll-like receptor-2 causes social deficits and memory impairment in female offspring | Y. Lee [108] | Exclude | Animal study |
| 108. Tryptophan Metabolism Along the Kynurenine Pathway Downstream of Toll-like Receptor Stimulation in Peripheral Monocytes | F. Orhan [109] | Exclude | Healthy subjects were recruited in this study |
| 109. Autoimmune diseases, gastrointestinal disorders and the microbiome in schizophrenia: more than a gut feeling | E. G. Severance [110] | Exclude | Review |
| 110. Experimental brain ischemic preconditioning: A concept to putative targets | A. Sharma [111] | Exclude | Review |
| 111. Erratum: Uniting the neurodevelopmental and immunological hypotheses: Neuregulin 1 receptor ERBB and Toll-like receptor activation in first-episode schizophrenia | S.Keri [77] | Exclude | Erratum (duplicate) |
| 112. Evidence for Decreased Density of Calretinin-Immunopositive Neurons in the Caudate Nucleus in Patients With Schizophrenia | I. Adorjan [112] | Exclude | No TLRs were measured |
| 113. Microbiome, inflammation, epigenetic alterations, and mental diseases | R. Alam [113] | Exclude | Review |
| 114. Sex-dependent effects of perinatal inflammation on the brain: Implication for neuro-psychiatric disorders | M. Ardalan [114] | Exclude | Review |
| 115. Pattern of expression of Toll like receptor (TLR)-3 and -4 genes in drug-naïve and antipsychotic treated patients diagnosed with schizophrenia | R. Balaji [115] | Include | * Independent data extraction (February 2024): M.J., R.S., S.B.  * Conflicts: M.A. and M.J. |
| 116. Traumagenics: At the intersect of childhood trauma, immunity and psychosis | KA. Chase [116] | Include | * Independent data extraction (February 2024): M.J., R.S., S.B.  * Conflicts: M.A. and M.J. |
| 117. From probiotics to psychobiotics - the gut-brain axis in psychiatric disorders | A. Zagorska [117] | Exclude | Review |
| 118. TLR/mTOR inflammatory signaling pathway: novel insight for the treatment of schizophrenia | N. A. Lashgari [118] | Exclude | Review |
| 119. The Emerging Role of Toll-Like Receptor-Mediated Neuroinflammatory Signals in Psychiatric Disorders and Acquired Epilepsy | A. Chaudhary [119] | Exclude | Review |
| 120. Association between genetic variants of TLR2, TLR4, TLR9 and schizophrenia | F. Ayari [120] | Exclude | Non-English |
| 121. Aberrant Histone Modification of TNFAIP3, TLR4, TNIP2, miR-146a, and miR-155 in Major Depressive Disorder | C. C. Tseng [121] | Exclude | Discussed just major depressive disorder |
| 122. Toll-Like Receptor (TLR) 1, 2, and 6 Gene Polymorphisms Support Evidence of Innate Immune Factors in Schizophrenia | C. E. Sotelo-Ramírez [122] | Exclude | TLR-4 was not measured |
| 123. Increased proportions of circulating proinflammatory monocytes and macrophages expressing toll‐like receptor 4 in individuals with schizophrenia and bipolar disorder receiving medication. | SY. Tsai [123] | Include | * Independent data extraction (February 2024): M.J., R.S., S.B.  * Conflicts: M.A. and M.J. |
| 124. Counting the Toll of Inflammation on Schizophrenia-A Potential Role for Toll-like Receptors | S. R. Patlola [124] | Exclude | Review |
| 125. Whole Genome Sequencing Revealed Inherited Rare Oligogenic Variants Contributing to Schizophrenia and Major Depressive Disorder in Two Families | I. H. Chung [125] | Exclude | Discussed just major depressive disorder |

3.**Reply**: Thank you very much for your precious comment. The flowchart of the study selection process is provided in **Figure 1**.

# **Reviewer 2:**

**This study addresses an important topic and has a suitable overall structure. However, it requires more clarity and detail in certain sections:**

7. **Reply**: Dear reviewer,

Thank you very much for your kind words and for providing us with the opportunity to strengthen our research. We sincerely appreciate your precious comments. Having carefully considered the comments and suggestions, we have made all the relevant changes to our manuscript as outlined below in an itemized, point-by-point manner. The revisions requested have been track-changed and highlighted within the manuscript in response.

## **1. Introduction**

1. **Please provide additional details about the selection of TLR4 as a specific receptor and its role in the pathophysiology of schizophrenia. Additionally, explain why this receptor is more significant compared to other immune pathways or molecules.**
   - **Reply**: Thank you for your valuable feedback. Based on your comment, we revised the third paragraph of the Introduction and included additional elaboration, along with appropriate references, to clarify the rationale behind the specific role of TLR4 in the pathophysiology of schizophrenia: “*Recent studies have suggested a diminished monocytic TLR4 response to LPS stimulation in individuals with schizophrenia, with increased TLR4 expression as a compensatory mechanism for the attenuated monocytic activation [77, 86]. Moreover, polymorphisms in the TLR4 gene have been shown to increase susceptibility to the disorder [56]. Postmortem studies have also revealed altered TLR4 expression and signaling in the brains of individuals with schizophrenia [107]. Altered TLR4 expression and function may contribute to schizophrenia pathogenesis by affecting innate immune system activation [107], neuroinflammation [126], increased susceptibility to infection [127, 128], and structural brain changes [129]. Higher monocytic TLR4 expression in people with schizophrenia [77, 86, 115, 130] may contribute to neuroinflammation [107, 126, 131]. Subsequently, the altered inflammatory response may contribute to the adverse effects of schizophrenia on the brain, including cognitive function [107, 132]. At the same time, while TLR4 might be overexpressed in persons with schizophrenia, its stimulation might induce a weaker increase in TLR4 expression and reduced proinflammatory cytokine production compared to healthy controls (HCs) [69, 77, 86, 131]. This suggests that individuals with schizophrenia may be less able to react appropriately to infections [127, 128], which could result in chronic inflammatory states that worsen symptoms of schizophrenia [131]*.”
2. **Elaborate further on the potential factors that might explain inconsistencies in previous study findings (e.g., differences in experimental methods, population characteristics, or medication effects).**
   - **Reply**: Thank you very much for your valuable feedback. According to your precious comment, the following sentence was added to the Introduction: “*Several factors may have contributed to these discrepancies, including variations in antipsychotic (AP) medication (type and dosage), treatment duration, and participant characteristics*.” A detailed explanation is provided in the second paragraph of the discussion to prevent the Introduction section from getting too long: “*Several factors might contribute to the discrepancies observed between the studies, including differences in the type or dosage of AP medication, therapy duration, and characteristics of study participants (e.g., disease duration and severity). These factors are plausible given their potential to influence an individual's immune and inflammatory status. For instance, a recent systematic review and meta-analysis revealed a significant reduction in pro-inflammatory cytokines following risperidone treatment, an effect not observed with clozapine (52). Subgroup analyses, comparing first-episode psychosis to chronic patients, demonstrated that disease duration also influenced the extent of cytokine alterations. Risperidone treatment significantly lowered levels of IL-6 and TNF-α in patients with chronic disease, while this effect was not evident in individuals experiencing their first episode of psychosis (52). Future investigations should encompass a broad range of medications, along with multiple follow-up assessments for a longer duration*”.
3. **Clearly specify how the findings of this systematic review can contribute to improving current therapeutic approaches or inform novel treatment strategies for schizophrenia.**
   - **Reply**: Thank you very much for your valuable comment. Accordingly, the following paragraph was added at the end of the Introduction section: “*The aim was to summarize existing literature, identify knowledge gaps, and focus on the dysregulated immune response, particularly involving TLR4, which may significantly contribute to the pathophysiology of schizophrenia. This review could pave the way for future research exploring potential avenues for novel therapeutic strategies. Such strategies may involve modulating TLR4 activity or targeting related downstream pathways as adjunctive treatments alongside standard antipsychotics*.”
   - Please note that since we added some information to the Introduction in the revised version, some previous sentences were omitted to prevent the introduction from being too long.

## **2. Methods**

1. **Please provide more details about the use of keyword combinations (e.g., Boolean logic such as AND/OR) and any filters applied during the search process.**
   - **Reply**: Thank you very much for your valuable comment. Our main research question was to investigate the basal expression and activation pattern of TLR4 in PBMCs, including monocytes, among individuals with schizophrenia compared to HCs. We conducted a systematic search in three databases, including MEDLINE (via PubMed), Scopus, and Web of Science. Then, we used the “Patient, Intervention, Comparison, and Outcome” (PICO) model to formulate our research question [1]:

- Patient: Persons with schizophrenia
- Intervention/exposure: not applicable
- Comparison: HCs
- Outcome(s):

1. TLR4 basal expression patterns in PBMCs

2. TLR4 activation patterns in PBMCs

Then, two PICO elements were used to convert our research question to a search strategy:

i. Patient-related MeSH Terms and text word terms: (“schizophrenia”, “schizophrenic”, “schizophrenias”, “psychosis”, and “psychotic”) AND
ii. Outcome-related MeSH terms and text word terms: “toll-like”, “toll-like receptor”, “toll-like receptor 4”, and “TLR”

All the information is written in the “Methods section > Objectives and review questions/ Study design, information sources, and search strategy”. **However, since we taught that providing the whole search strategy within the manuscript text might be space-consuming, we provided the detailed search strategy in Supplemental Tables S3-S5**. Sincerely, please let us know if any other clarifications are required considering the search strategy. Additionally, according to your comment, we also added that: “*No filter was applied during the search process*”.

| **MEDLINE (via PubMed)** | No. |
| --- | --- |
| 3. (Toll-like receptors [MeSH Terms] OR “toll-like receptors” [Title/Abstract] OR TLR [Title/Abstract] OR “toll-like receptor” [Title/Abstract] OR “Toll-like” [Title/Abstract] OR “Toll-Like receptor 4” [Title/Abstract] OR TLR4 [Title/Abstract] OR TLR-4 [Title/Abstract] OR “Toll-Like receptor 4” [MeSH Terms]) | **73579** |
| 4. (Schizophrenia [MeSH Terms] OR schizophrenia [Title/Abstract] OR psychotic [Title/Abstract] OR psychosis [Title/Abstract] OR psychotic disorders [MeSH Terms] OR “psychotic disorders” [Title/Abstract] OR “psychotic disorder” [Title/Abstract] OR schizophrenias [Title/Abstract] OR schizophrenic [Title/Abstract]) | **228052** |
| #1 AND #2 | **112** |
| **Web of Science** |  |
| 3. (TS= (“Toll-like receptors”) OR TS= (“toll-like receptor”) OR TS= (TLR) OR TS= (“Toll-like”) OR TS= (“Toll-Like receptor 4”) OR TS= (TLR4) OR TS= (TLR-4)) | **87616** |
| 4. (TS= (Schizophrenia) OR TS= (psychotic) OR TS= (psychosis) OR TS= (“psychotic disorders”) OR TS= (“psychotic disorder”) OR TS= (schizophrenias) OR TS= (schizophrenic)) | **266798** |
| #1 AND #2 | **171** |
| **Scopus** |  |
| 3. (INDEXTERMS("Toll-like receptors") OR TITLE-ABS("toll-like receptors") OR TITLE-ABS(TLR) OR TITLE-ABS("toll-like receptor") OR TITLE-ABS(Toll-like) OR TITLE-ABS("Toll-Like receptor 4") OR TITLE-ABS(TLR4) OR TITLE-ABS(TLR-4) OR INDEXTERMS("Toll-Like receptor 4")) | **111233** |
| 4. (TITLE-ABS-KEY (Schizophrenia ) OR TITLE-ABS-KEY (psychotic ) OR TITLE-ABS-KEY (psychosis ) OR TITLE-ABS-KEY ("psychotic disorders" ) OR TITLE-ABS-KEY ("psychotic disorder" ) OR TITLE-ABS-KEY (schizophrenias ) OR TITLE-ABS-KEY (schizophrenic )) | **340059** |
| #1 AND #2 | **339** |

2. **Clarify the number of studies retrieved at each stage of the search process and provide transparent reasons for their exclusion.**
   - **Reply**: Thank you very much for your thoughtful feedback. First, a total of 622 records from the three databases were initially imported into EndNote software (version X9, Clarivate Plc). Then, duplicates were removed, yielding 452 records for initial screening. Then, two independent researchers (R.S. and S.B.) screened all the 452 records using titles and abstracts. Among these articles, 125 potentially eligible articles remained for full-text screening by three independent authors (M.J., S.B., and R.S.), including one expert author (M.J.). Notably, the references of these articles were also manually searched for any potentially related article that was not retrieved initially by database searching (Hand searching method). The records that did not align with the predefined eligibility criteria were excluded. In cases of discrepancies, conflicts were resolved by reaching a consensus among all authors (including the two corresponding authors).

- Among these 125 articles, 114 did not align with the predefined eligibility criteria, leaving 11 original articles aligning with the research question. **Figure 1** shows the selection process and the number of studies retrieved at each stage of the search process. **The reason for the exclusion of each study is reported in the revised version of the manuscript as Supplementary Table S6.** The information regarding the selection of studies is reported in the “Methods section > Selection process”, as well as the “Results section > Literature search and study selection”. According to your valuable comment, to ensure integrity, completeness, and logic behind selecting the references, the table below also shows the reason(s) for excluding each single study. Please let us know if there are any other clarifications required concerning selecting the studies.

- Notably, since many of the studies had more than one reason for exclusion, in addition to adding Supplementary Table S6, we revised the manuscript to provide a clearer overview of the reasons behind excluding the studies. For studies with more than one reason, we reported the “main” reason in the text (other reasons are reported in **Supplementary Table S6**).

**Table S6. Studies identified in the literature search that proceeded to full-text screening**

| **Title** | **First author** | **Eligibility** | **Exclusion reason/ Extractors^[2]^** |
| --- | --- | --- | --- |
| 126. JNK signaling mediates aspects of maternal immune activation: importance of maternal genotype in relation to schizophrenia risk | R.L Openshow [2] | Exclude | Animal study |
| 127. CNS Macrophages and Infant Infections | A. Oschwald [3] | Exclude | Review |
| 128. Role of inflammation in epilepsy and neurobehavioral comorbidities: Implication for therapy | Y.N. Paudel [4] | Exclude | Review |
| 129. The role of the gut microbiota in the pathophysiology of mental and neurological disorders | M.M. Pusceddu [5] | Exclude | Review |
| 130. N-3 polyunsaturated fatty acids and clozapine abrogates poly I: C-induced immune alterations in primary hippocampal neurons | B.M.M. Ribeiro [6] | Exclude | In-vitro |
| 131. Lack of Helios During Neural Development Induces Adult Schizophrenia-Like Behaviors Associated With Aberrant Levels of the TRIF-Recruiter Protein WDFY1 | A. Sancho-Balsells [7] | Exclude | Animal study |
| 132. Targeting the NLRP3 Inflammasome-Related Pathways via Tianeptine Treatment-Suppressed Microglia Polarization to the M1 Phenotype in Lipopolysaccharide-Stimulated Cultures | J. Slusarczyk [8] | Exclude | In-vitro |
| 133. Importance of the immune system in mediating plasticity of the brain and behavior | L. Tian [9] | Exclude | Review |
| 134. Evidence of microglial activation following exposure to serum from first-onset drug-naïve schizophrenia patients | G.F.V. Rees [10] | Exclude | levels of TLR were not discussed. |
| 135. CD157 and Brain Immune System in (Patho)physiological Conditions: Focus on Brain Plasticity | O.L. Lopatina [11] | Exclude | Review |
| 136. Neuroinflammation in the dorsolateral prefrontal cortex in elderly chronic schizophrenia | I. Lopez-Gonzalez [12] | Exclude | Post-mortem study |
| 137. The Role of Immune Factors in Shaping Fetal Neurodevelopment | A. Lu-Culligan [13] | Exclude | Review |
| 138. Differential regulation of the TLR4 signaling pathway in post-mortem prefrontal cortex and cerebellum in chronic schizophrenia: Relationship with SP transcription factors | K.S. MacDowell [14] | Exclude | Post-mortem study |
| 139. Immunomodulatory effects of antipsychotic treatment on gene expression in first-episode psychosis | O. Mantere [15] | Exclude | Discussed psychosis, not schizophrenia specifically |
| 140. Emerging role of innate B1 cells in the pathophysiology of autoimmune and neuroimmune diseases: Association with inflammation, oxidative and nitrosative stress and autoimmune responses | G. Morris [16] | Exclude | Review |
| 141. Active psychosis and pro-inflammatory cytokines in first episode of psychosis | G. Pardo-de-Santayana [17] | Exclude | TLR was not discussed, cytokines were investigated |
| 142. Anxiety-like behavior and microglial activation in the amygdala after acute neuroinflammation induced by microbial neuraminidase | A. Leon-Rodriguez [18] | Exclude | Animal study |
| 143. Association between TLR2 polymorphisms (- 196-174 Ins/Del, R677W, R753Q, and P631H) and schizophrenia in a Tunisian population | Y. Aflouk [19] | Exclude | Levels of TLR were not discussed, TLR2 polymorphisms were only discussed |
| 144. Association of complement component 4 with neuroimmune abnormalities in the subventricular zone in schizophrenia and autism spectrum disorders | T. C. M. Mou [20] | Exclude | No TLR was discussed, and complement component 4 was explained |
| 145. The association of inflammatory status and immunological parameters with single-nucleotide polymorphisms of cytokine and Toll-like receptor genes in patients with schizophrenia | I. K. Malashenkova [21] | Exclude | No levels of TLR were discussed, TLR polymorphisms were only discussed |
| 146. Association of TLR-4 896A/G, TLR-4 1196C/T, and TLR-9 C/T polymorphism with schizophrenia in Indian Bengalee patient | J. Gurung [22] | Exclude | No levels of TLR were discussed, TLR polymorphisms were only discussed |
| 147. Association of toll-like receptor 2 gene polymorphism (rs3804099) with susceptibility to Schizophrenia risk in the Dogra population of Jammu region, North India | I. Sharma [23] | Exclude | No levels of TLR were discussed, TLR polymorphisms were only discussed |
| 148. Astrocyte Bioenergetics and Major Psychiatric Disorders | I. V. Maly [24] | Exclude | Book Series |
| 149. Charting the proteome landscape in major psychiatric disorders: From biomarkers to biological pathways towards drug discovery | Fern, B. S. es [25] | Exclude | systematic review and comprehensive *in silico* analysis |
| 150. Cognitive impairment in psychiatric diseases: Biomarkers of diagnosis, treatment, and prevention | Y. Wang [26] | Exclude | Review |
| 151. Consequences of Viral Infection and Cytokine Production During Pregnancy on Brain Development in Offspring | D. Elgueta [27] | Exclude | Review |
| 152. Differential Effects of Toll-Like Receptor Activation and Differential Mediation by MAP Kinases of Immune Responses in Microglial Cells | J. Kwon, C. Arsenis [28] | Exclude | In-vitro |
| 153. Dysfunctional monocytic toll-like receptor 4 signaling pathway and cognitive deficits in chronic schizophrenia patients with tardive dyskinesia | N. Li [29] | Include | * Independent data extraction (February 2024): M.J., R.S., S.B.  * Conflicts: M.A. and M.J. |
| 154. Early life Adversity, functional connectivity and cognitive performance in Schizophrenia: The mediating role of IL-6 | S. King [30] | Exclude | No levels of TLR were discussed |
| 155. Editorial: Cognitive Dysfunctions in Psychiatric Disorders: Brain-Immune Interaction Mechanisms and Integrative Therapeutic Approaches | H. Xu [31] | Exclude | Editorial |
| 156. Editorial: The inflammation markers in schizophrenia and bipolar disorder: Do we have promising results? | A. Arslan [32] | Exclude | Editorial |
| 157. Environmental Risk Factors for Schizophrenia and Bipolar Disorder and Their Relationship to Genetic Risk: Current Knowledge and Future Directions | N. Robinson [33] | Exclude | Review |
| 158. First Episode Psychosis and Schizophrenia Are Systemic Neuro-Immune Disorders Triggered by a Biotic Stimulus in Individuals with Reduced Immune Regulation and Neuroprotection | M. Maes [34] | Exclude | Secondary analysis, no data on TLR |
| 159. Gut microbiota in psychiatric disorders: Better understanding or more complexity to be resolved? | B. Misiak [35] | Exclude | Editorial |
| 160. Human endogenous retrovirus W family envelope protein (HERV-W env) facilitates the production of TNF-α and IL-10 by inhibiting MyD88s in glial cells | X. Wang [36] | Exclude | In-vitro |
| 161. Identifying crucial biomarkers in peripheral blood of schizophrenia and screening therapeutic agents by comprehensive bioinformatics analysis | M. Xie [37] | Exclude | Secondary analysis, no distinct data on the levels of TLR |
| 162. Immune System Abnormalities in Schizophrenia: An Integrative View and Translational Perspectives | E. Ermakov [38] | Exclude | Review |
| 163. Impact of SARS-CoV-2 on Host Factors Involved in Mental Disorders | R. Rhoades [39] | Exclude | Review |
| 164. Influenza Virus Infection during Pregnancy as a Trigger of Acute and Chronic Complications | O. Oseghale [40] | Exclude | Review |
| 165. Involvement of TCF7L2 in generation of morphine-induced antinociceptive tolerance and hyperalgesia by modulating TLR4/ NF-κB/NLRP3 in microglia | J. Chen [41] | Exclude | Animal study |
| 166. A loss of mature microglial markers without immune activation in schizophrenia | G. J. L. J. Snijders [42] | Exclude | Review |
| 167. Maternal inflammation and its ramifications on fetal neurodevelopment | H.K. Kwon [43] | Exclude | Review |
| 168. Modulatory Effect of Gut Microbiota on the Gut-Brain, Gut-Bone Axes, and the Impact of Cannabinoids | I. Ibrahim [44] | Exclude | Review |
| 169. Negative Regulation of the IL-1 System by IL-1R2 and IL-1R8: Relevance in Pathophysiology and Disease | D. Supino [45] | Exclude | Review |
| 170. Neurobiological Highlights of Cognitive Impairment in Psychiatric Disorders | A. Morozova [46] | Exclude | Review |
| 171. Peripheral NF-κB dysregulation in people with schizophrenia drives inflammation: putative anti-inflammatory functions of NF-κB kinases | C.E. Murphy [47] | Exclude | Evaluating TLR4 gene expression in peripheral blood leukocytes (not inclusively in PBMC) |
| 172. A Possible Causal Involvement of Neuroinflammatory, Purinergic P2X7 Receptors in Psychiatric Disorders | Y. Zhang [48] | Exclude | Review |
| 173. Potential Neurocognitive Symptoms Due to Respiratory Syncytial Virus Infection | C.A. Andrade [49] | Exclude | Review |
| 174. Psychosis symptoms following aberrant immunity in the brain | A. Ozaki [50] | Exclude | Editorial |
| 175. The relationship between TLR4/NF-κB/IL-1β signaling, cognitive impairment, and white-matter integrity in patients with stable chronic schizophrenia | H. Li [51] | Included | * Independent data extraction (February 2024): M.J., R.S., S.B.  * Conflicts: M.A. and M.J. |
| 176. The role of the gut microbiome in the development of schizophrenia | J.R. Kelly [52] | Exclude | Review |
| 177. Schizophrenia Hypothesis: Autonomic Nervous System Dysregulation of Fetal and Adult Immune Tolerance | T. Carnac [53] | Exclude | Review |
| 178. Targeting s100b protein as a surrogate biomarker and its role in various neurological disorders | U. Langeh [54] | Exclude | Review |
| 179. TOLL-LIKE RECEPTOR (TLR) PATHWAY EXPRESSION IN DORSOLATERAL PREFRONTAL (BA46) AND ORBITOFRONTAL (BA11) CORTICES IN SCHIZOPHRENIA AND MOOD DISORDER | T. Ketharanathan [55] | Exclude | Post-mortem study on schizophrenia |
| 180. Toll-Like Receptor 4 Gene Polymorphisms and Susceptibility to Schizophrenia: A Case-Control Study | M. Mostafa [56] | Exclude | No levels of TLR were discussed, TLR polymorphisms were only discussed |
| 181. Virus-Induced Maternal Immune Activation as an Environmental Factor in the Etiology of Autism and Schizophrenia | A. Massrali [57] | Exclude | Review |
| 182. Antipsychotics influence Toll-like receptor (TLR) expression and its relationship with cognitive functions in schizophrenia | S. Keri [58] | Include | * Independent data extraction (February 2024): M.J., R.S., S.B.  * Conflicts: M.A. and M.J. |
| 183. Uniting the neurodevelopmental and immunological hypotheses: Neuregulin 1 receptor ErbB and Toll-like receptor activation in first-episode schizophrenia | S. Keri [59] | Exclude | Erratum |
| 184. The expression of toll-like receptors in peripheral blood mononuclear cells is altered in schizophrenia | E. Kozlowska [60] | Include | * Independent data extraction (February 2024): M.J., R.S., S.B.  * Conflicts: M.A. and M.J. |
| 185. Oxidative stress and mitochondrial dysfunction in human diseases: Pathophysiology, predictive biomarkers, therapeutic | C.J. Li [61] | Exclude | Editorial |
| 186. TLR4 (Toll-like receptor-4) expression and frontal-cingulate volumes in schizophrenia | H. Li [62] | Exclude | Non-English |
| 187. The TMEM106B FTLD-protective variant, rs1990621, is also associated with increased neuronal proportion | Z. Li [63] | Exclude | No TLRs were measured |
| 188. Maternal Murine Cytomegalovirus Infection during Pregnancy Up-regulates the Gene Expression of Toll-like Receptor 2 and 4 in Placenta | Y. Liao [64] | Exclude | Animal study |
| 189. Periodontal pathogens and neuropsychiatric health | A. Wadhawan [65] | Exclude | Review |
| 190. Human endogenous retroviral envelope protein Syncytin-1 and inflammatory abnormalities in neuropsychological diseases | X. Wang [66] | Exclude | Review |
| 191. Syncytin-1, an endogenous retroviral protein, triggers the activation of CRP via TLR3 signal cascade in glial cells | X. Wang [67] | Exclude | In-vitro |
| 192. Longitudinal Analyses of Blood Transcriptome During Conversion to Psychosis | B. Chaumette [68] | Exclude | No TLRs were measured |
| 193. More dampened monocytic Toll-like receptor 4 response to lipopolysaccharide and its association with cognitive function in Chinese Han first-episode patients with schizophrenia | S. Chen [69] | Include | * Independent data extraction (February 2024): M.J., R.S., S.B.  * Conflicts: M.A. and M.J. |
| 194. Natural Catalytic IgGs Hydrolyzing Histones in Schizophrenia: Are They the Link between Humoral Immunity and Inflammation? | E.A. Ermakov [70] | Exclude | No TLRs were measured |
| 195. Neural cell responses upon exposure to human endogenous retroviruses | J. Gruchot [71] | Exclude | Review |
| 196. Neuroimmune biomarkers in mental illness | J.W. Herron [72] | Exclude | Review |
| 197. Microbiota-immune system interactions in human neurological disorders | Q. Huang [73] | Exclude | Review |
| 198. Expression and Functionality Study of 9 Toll-Like Receptors in 33 Drug-Naive Non-Affective First Episode Psychosis Individuals: A 3-Month Study | M. Juncal-Ruiz [74] | Exclude | Discussed psychosis, not schizophrenia specifically |
| 199. Cross talk: The microbiota and neurodevelopmental disorders | J.R. Kelly [75] | Exclude | Review |
| 200. Erratum: Uniting the neurodevelopmental and immunological hypotheses: Neuregulin 1 receptor ErbB and Toll-like receptor activation in first-episode schizophrenia | S. Keri [76] | Exclude | Erratum |
| 201. Uniting the neurodevelopmental and immunological hypotheses: Neuregulin 1 receptor ErbB and Toll-like receptor activation in first-episode schizophrenia | S. Keri [77] | Include | * Independent data extraction (February 2024): M.J., R.S., S.B.  * Conflicts: M.A. and M.J. |
| 202. The role of cytokines in mediating effects of prenatal infection on the fetus: implications for schizophrenia | H. Ashdown [78] | Exclude | Review |
| 203. Neonatal polyI:C treatment in mice results in schizophrenia-like behavioral and neurochemical abnormalities in adulthood | D. Ibi [79] | Exclude | Animal study |
| 204. Expression of anti-cardiolipin antibodies and inflammatory associated factors in patients with schizophrenia | SH. Chang [80] | Include | * Independent data extraction (February 2024): M.J., R.S., S.B.  * Conflicts: M.A. and M.J. |
| 205. ALTERED TOLL-LIKE RECEPTOR RESPONSES IN SCHIZOPHRENIA AND BIPOLAR DISORDER PATIENTS | D.P. McKernan [81] | Exclude | Review |
| 206. Enhanced peripheral toll-like receptor responses in psychosis: further evidence of a pro-inflammatory phenotype | D. P. McKernan [82] | Exclude | TLR-4 was not measured |
| 207. Cellular and Molecular Mechanisms of Autoimmune Disease | B. Bolon [83] | Exclude | Review |
| 208. Monocytic HLA DR antigens in schizophrenic patients | D. Krause [84] | Exclude | TLR-4 was not measured |
| 209. Toll-like receptor 2 polymorphisms are associated with poor concentration in schizophrenia patients in a Korean population | S. M. Lee [85] | Exclude | TLR-4 was not measured |
| 210. Impaired monocyte activation in schizophrenia | N. Müller [86] | Include | * Independent data extraction (February 2024): M.J., R.S., S.B.  * Conflicts: M.A. and M.J. |
| 211. Possible involvement of TLRs and hemichannels in stress-induced CNS dysfunction via mastocytes, and glia activation | A. Aguirre [87] | Exclude | Review |
| 212. Pathophysiology of bacterial infection of the central nervous system and its putative role in the pathogenesis of behavioral changes | T. Barichello [88] | Exclude | Review |
| 213. Oligodendroglial Alterations and the Role of Microglia in White Matter Injury: Relevance to Schizophrenia | L. J. Chew [89] | Exclude | Review |
| 214. Neuroinflammation in mood disorders: Mechanisms and drug targets | A. T. Hopper [90] | Exclude | Review |
| 215. Association between genetic polymorphisms of Toll-like receptor 2 (TLR2) and schizophrenia in the Korean population | W. S. Kang [91] | Exclude | TLR-4 was not measured |
| 216. TLR7 Negatively Regulates Dendrite Outgrowth through the Myd88-c-Fos-IL-6 Pathway | H. Y. Liu [92] | Exclude | Animal study and in-vitro |
| 217. Role of the Toll Like receptor (TLR) radical cycle in chronic inflammation: possible treatments targeting the TLR4 pathway | K. Lucas [93] | Exclude | Review |
| 218. Innate immunity and neuroinflammation | A. Shastri [94] | Exclude | Review |
| 219. Intermittent or sustained systemic inflammation and the preterm brain | O. Dammann [95] | Exclude | Review |
| 220. Sarm1 deficiency impairs synaptic function and leads to behavioral deficits, which can be ameliorated by an mGluR allosteric modulator | C. W. Lin [96] | Exclude | No TLR were measured |
| 221. Effects of Genetic and Environmental Factors on Neuropsychological Development | T. Nagai [97] | Exclude | Review |
| 222. Influenza vaccination during early pregnancy contributes to neurogenesis and behavioral function in offspring | Y. C. Xia [98] | Exclude | Animal study |
| 223. Protein-interaction-network-based analysis for genome-wide association analysis of schizophrenia in Han Chinese population | H. Yu [99] | Exclude | No TLR were measured |
| 224. A molecular pathway analysis informs the genetic background at risk for schizophrenia | C. Crisafulli [100] | Exclude | This article discussed molecular pathways and no TLR were measured. |
| 225. Innate immune response is differentially dysregulated between bipolar disease and schizophrenia | A. de Baumont [101] | Exclude | No TLR were measured |
| 226. Psychedelics and Immunomodulation: Novel Approaches and Therapeutic Opportunities | A. Szabo [102] | Exclude | Review |
| 227. Commentary: Maternal immune activation evoked by polyinosinic: Polycytidylic acid does not evoke microglial cell activation in the embryo | H. G. Bernstein [103] | Exclude | Commentary |
| 228. The Microbiota and Gut-Brain Axis: Contributions to the Immunopathogenesis of Schizophrenia | J. R. Caso [104] | Exclude | Review |
| 229. Interactions between the gut microbiome and the central nervous system and their role in schizophrenia, bipolar disorder and depression | A. A. Chrobak [105] | Exclude | Review |
| 230. Innate immune receptor Toll-like receptor 4 signaling in neuropsychiatric diseases | B. García Bueno [106] | Exclude | Review |
| 231. Evidence of activation of the Toll-like receptor-4 proinflammatory pathway in patients with schizophrenia | B. García-Bueno [107] | Exclude | Post-mortem study |
| 232. Activation of maternal toll-like receptor-2 causes social deficits and memory impairment in female offspring | Y. Lee [108] | Exclude | Animal study |
| 233. Tryptophan Metabolism Along the Kynurenine Pathway Downstream of Toll-like Receptor Stimulation in Peripheral Monocytes | F. Orhan [109] | Exclude | Healthy subjects were recruited in this study |
| 234. Autoimmune diseases, gastrointestinal disorders and the microbiome in schizophrenia: more than a gut feeling | E. G. Severance [110] | Exclude | Review |
| 235. Experimental brain ischemic preconditioning: A concept to putative targets | A. Sharma [111] | Exclude | Review |
| 236. Erratum: Uniting the neurodevelopmental and immunological hypotheses: Neuregulin 1 receptor ERBB and Toll-like receptor activation in first-episode schizophrenia | S. Keri [77] | Exclude | Erratum (duplicate) |
| 237. Evidence for Decreased Density of Calretinin-Immunopositive Neurons in the Caudate Nucleus in Patients With Schizophrenia | I. Adorjan [112] | Exclude | No TLRs were measured |
| 238. Microbiome, inflammation, epigenetic alterations, and mental diseases | R. Alam [113] | Exclude | Review |
| 239. Sex-dependent effects of perinatal inflammation on the brain: Implication for neuro-psychiatric disorders | M. Ardalan [114] | Exclude | Review |
| 240. Pattern of expression of Toll like receptor (TLR)-3 and -4 genes in drug-naïve and antipsychotic treated patients diagnosed with schizophrenia | R. Balaji [115] | Include | * Independent data extraction (February 2024): M.J., R.S., S.B.  * Conflicts: M.A. and M.J. |
| 241. Traumagenics: At the intersect of childhood trauma, immunity and psychosis | KA. Chase [116] | Include | * Independent data extraction (February 2024): M.J., R.S., S.B.  * Conflicts: M.A. and M.J. |
| 242. From probiotics to psychobiotics - the gut-brain axis in psychiatric disorders | A. Zagorska [117] | Exclude | Review |
| 243. TLR/mTOR inflammatory signaling pathway: novel insight for the treatment of schizophrenia | N. A. Lashgari [118] | Exclude | Review |
| 244. The Emerging Role of Toll-Like Receptor-Mediated Neuroinflammatory Signals in Psychiatric Disorders and Acquired Epilepsy | A. Chaudhary [119] | Exclude | Review |
| 245. Association between genetic variants of TLR2, TLR4, TLR9 and schizophrenia | F. Ayari [120] | Exclude | Non-English |
| 246. Aberrant Histone Modification of TNFAIP3, TLR4, TNIP2, miR-146a, and miR-155 in Major Depressive Disorder | C. C. Tseng [121] | Exclude | Discussed just major depressive disorder |
| 247. Toll-Like Receptor (TLR) 1, 2, and 6 Gene Polymorphisms Support Evidence of Innate Immune Factors in Schizophrenia | C. E. Sotelo-Ramírez [122] | Exclude | TLR-4 was not measured |
| 248. Increased proportions of circulating proinflammatory monocytes and macrophages expressing toll‐like receptor 4 in individuals with schizophrenia and bipolar disorder receiving medication. | SY. Tsai [123] | Include | * Independent data extraction (February 2024): M.J., R.S., S.B.  * Conflicts: M.A. and M.J. |
| 249. Counting the Toll of Inflammation on Schizophrenia-A Potential Role for Toll-like Receptors | S. R. Patlola [124] | Exclude | Review |
| 250. Whole Genome Sequencing Revealed Inherited Rare Oligogenic Variants Contributing to Schizophrenia and Major Depressive Disorder in Two Families | I. H. Chung [125] | Exclude | Discussed just major depressive disorder |

3. **Explain why certain studies were categorized as “inconsistent with the research question.”**
   - **Reply**: Thank you very much for your thoughtful feedback. We understand that the phrase “inconsistent with the research question” seems too general and does not convey the exact reason(s) for the exclusion of each study, hence adding **Supplementary Table S6** with detailed information on the reason(s) behind excluding each study. We also provided clearer information regarding the exclusion reasons in the Methods section: “*Hence, 125 articles remained for full-text screening, 114 of which were excluded for the following reasons: not evaluating TLR4-related outcomes (n=24), evaluating outcomes in populations other than people with schizophrenia (n=5), being non-original articles (n=64), animal studies (n=9), in vitro studies (n=5), post-mortem studies (n=4), or non-English studies (n=2). Additionally, one study was excluded for evaluating TLR4 gene expression in peripheral blood leukocytes rather than specifically in PBMCs (44)*”. (The reason that some of the numbers are different from the previous version is that many articles had more than one reason for exclusion and here we only reported the most “general” reason for their exclusion. However, the table provides a more detailed view.)
   - For instance, the study population in some studies was not consistent with our research population (people with schizophrenia):

| Immunomodulatory effects of antipsychotic treatment on gene expression in first-episode psychosis | O. Mantere [15] | Discussed psychosis, not schizophrenia specifically |
| --- | --- | --- |
| Expression and Functionality Study of 9 Toll-Like Receptors in 33 Drug-Naive Non-Affective First Episode Psychosis Individuals: A 3-Month Study | M. Juncal-Ruiz [74] | Discussed psychosis, not schizophrenia specifically |
| Tryptophan Metabolism Along the Kynurenine Pathway Downstream of Toll-like Receptor Stimulation in Peripheral Monocytes | F. Orhan [109] | Healthy subjects were recruited in this study |
| Aberrant Histone Modification of TNFAIP3, TLR4, TNIP2, miR-146a, and miR-155 in Major Depressive Disorder | C. C. Tseng [121] | Discussed just major depressive disorder |

- Some other studies assessed a different outcome regarding TLR(s), like TLR “polymorphism”.

| Association between TLR2 polymorphisms (- 196-174 Ins/Del, R677W, R753Q, and P631H) and schizophrenia in a Tunisian population | Y. Aflouk [19] | Levels of TLR were not discussed, TLR2 polymorphisms were only discussed |
| --- | --- | --- |
| The association of inflammatory status and immunological parameters with single-nucleotide polymorphisms of cytokine and Toll-like receptor genes in patients with schizophrenia | I. K. Malashenkova [21] | No levels of TLR were discussed, TLR polymorphisms were only discussed |
| Association of TLR-4 896A/G, TLR-4 1196C/T, and TLR-9 C/T polymorphism with schizophrenia in Indian Bengalee patient | J. Gurung [22] | No levels of TLR were discussed, TLR polymorphisms were only discussed |
| Association of toll-like receptor 2 gene polymorphism (rs3804099) with susceptibility to Schizophrenia risk in the Dogra population of Jammu region, North India | I. Sharma [23] | No levels of TLR were discussed, TLR polymorphisms were only discussed |

- Some studies evaluated TLR4 gene expression in peripheral blood leukocytes (not inclusively in PBMC)

| Peripheral NF-κB dysregulation in people with schizophrenia drives inflammation: putative anti-inflammatory functions of NF-κB kinases | C.E. Murphy [47] | Evaluating TLR4 gene expression in peripheral blood leukocytes (not inclusively in PBMC) |
| --- | --- | --- |

- Many studies did not measure TLR4-related outcomes. However, these studies were not excluded initially based on their title or abstract alone; instead, they were retained until the full text was reviewed to ensure no potentially eligible study was inadvertently excluded.

| Evidence of microglial activation following exposure to serum from first-onset drug-naïve schizophrenia patients | G.F.V. Rees [10] | levels of TLR were not discussed. |
| --- | --- | --- |
| Active psychosis and pro-inflammatory cytokines in first episode of psychosis | G. Pardo-de-Santayana [17] | TLR was not discussed, cytokines were investigated |
| Early life Adversity, functional connectivity and cognitive performance in Schizophrenia: The mediating role of IL-6 | S. King [30] | No levels of TLR were discussed |
| First Episode Psychosis and Schizophrenia Are Systemic Neuro-Immune Disorders Triggered by a Biotic Stimulus in Individuals with Reduced Immune Regulation and Neuroprotection | M. Maes [34] | Secondary analysis, no data on TLR |
| The TMEM106B FTLD-protective variant, rs1990621, is also associated with increased neuronal proportion | Z. Li [63] | No TLRs were measured |

## **3. Results**

1. **Please explain how the high heterogeneity of the studies (I² > 75%) impacts the interpretation of the results.**
   - **Reply**: We appreciate the reviewer’s observation regarding the high heterogeneity (I² > 75%) observed in our meta-analyses and the potential implications for the interpretation of the results. Below, we provide a detailed explanation to address these concerns.
a. ***Understanding Heterogeneity in Context:***
   - The high heterogeneity in our meta-analyses reflects the variability across studies in terms of effect sizes, methodologies, sample characteristics, and potential confounding factors such as differences in antipsychotic (AP) medication status, demographic variations, and study designs. Specifically, the I² statistics (e.g., 87.3% in Monocytes expressing TLR-4 and 68.9% in TLR-4 density on monocyte membranes) indicate that a substantial portion of the variability in the observed effect sizes can be attributed to between-study differences rather than sampling error.
   - It is critical to note that high heterogeneity is not inherently problematic but requires careful interpretation. Our use of a random-effects model with REML estimation is specifically designed to account for such variability, as it assumes that the true effect size may vary across studies and provides more conservative estimates, reducing the likelihood of overgeneralizing results.
b. ***Impact of Heterogeneity on Interpretation:***
   - The substantial heterogeneity observed does not undermine the validity of our findings but highlights the complex interplay of factors influencing TLR-4 expression in schizophrenia. The observed non-significant pooled effect sizes across AP-free and On-AP subgroups (e.g., -0.399 for Monocytes expressing TLR-4 and -0.317 for TLR density on monocyte membranes) suggest that while there is variability among studies, the overall trend remains consistent and robust.
   - Furthermore, our subgroup analyses based on AP status reveal that heterogeneity is largely driven by differences within the AP-free and On-AP groups rather than systematic biases. For example, in the Monocytes expressing TLR-4 meta-analysis, the Q statistics for AP-free (Q = 14.81, p < 0.001) and On-AP (Q = 24.67, p < 0.001) groups indicate significant variability within these subgroups, likely due to differences in treatment duration, dosage, and underlying pathophysiology of schizophrenia.
c. ***Steps Taken to Address Heterogeneity:***
   - Subgroup analyses: By stratifying studies into AP-free and On-AP subgroups, we minimized potential confounding effects related to medication status, allowing for a more nuanced understanding of TLR-4 expression dynamics.
   - Sensitivity analyses: Iterative removal of individual studies revealed that the overall findings were not significantly altered, underscoring the robustness of our results despite the presence of heterogeneity. For example, in the TLR density meta-analysis, excluding the study by Chen (2023) resulted in a statistically significant effect size (-0.469), suggesting that certain studies may disproportionately contribute to variability.
   - Publication bias assessment: The absence of significant publication bias, as demonstrated by Begg’s test and funnel plot analyses, further validates the reliability of our findings.
d. ***Biological and Clinical Implications of Heterogeneity:***
   - The high heterogeneity likely reflects real-world variations in biological responses to schizophrenia and its treatment, emphasizing the need for individualized approaches in research and clinical practice.
   - While high heterogeneity poses challenges for interpretation, it does not invalidate our results. Instead, it underscores the complexity of TLR-4 expression in schizophrenia and highlights the importance of using advanced statistical methods, such as random-effects models, to derive meaningful conclusions.
   - We hope this detailed explanation satisfactorily addresses the reviewer’s concern and demonstrates the robustness and validity of our approach and findings. According to your valuable comment, the following paragraph, summarizing the importance of interpreting the results while considering heterogeneity, was added to the Limitation section: “*Finally, the high heterogeneity observed in several meta-analyses (I² > 75%), particularly within subgroups stratified by AP status poses challenges in clearly interpreting the results (70). This variability likely arises from differences in study populations, methodologies, and treatment protocols. To address this, we employed a random-effects model with REML estimation to account for between-study variability, conducted subgroup analyses based on AP status to minimize confounding effects, and performed sensitivity analyses, which demonstrated the stability of our findings despite the heterogeneity. Additionally, publication bias assessments confirmed the robustness of our results*.”

## **4. Discussion**

1. **Highlight more explicitly how the findings of this study relate to practical applications or the development of new treatments for schizophrenia.**
   - **Reply**: Thank you very much for your insightful comment. According to your valuable comment, we added the following paragraph at the end of the Discussion section under the subheading of “Practical implications”: “*The study findings revealed a trend towards elevated monocytic TLR4 density alongside diminished monocytic TLR4 activation in individuals with schizophrenia, indicating a dysregulated immune response that may play a crucial role in the pathophysiology of the disorder. These findings open avenues for novel therapeutic strategies. Modulating TLR4 activity or targeting related downstream pathways through adjunctive therapies alongside standard antipsychotics holds promise for improving treatment outcomes. This is particularly relevant considering the potential link between TLR4 signaling and cognitive deficits, a critical and often challenging aspect of schizophrenia treatment.*”
2. **Provide further explanations about the potential factors contributing to inconsistencies in previous studies, including the effects of antipsychotic drugs, demographic differences, or variations in experimental methods. Suggestion: Addressing these points will significantly enhance the quality and impact of the manuscript, establishing it as a reliable and valuable resource in this field.**
   - **Reply**: Thank you very much for your constructive feedback. According to your mention, we discussed the possible reason for the observed inconsistencies between the study findings in the second paragraph of the Discussion: “*Several factors might contribute to the discrepancies observed between the studies, including differences in the type or dosage of AP medication, therapy duration, and characteristics of study participants (e.g., disease duration and severity). These factors are plausible given their potential to influence an individual's immune and inflammatory status. For instance, a recent systematic review and meta-analysis revealed a significant reduction in pro-inflammatory cytokines following risperidone treatment, an effect not observed with clozapine [133]. Subgroup analyses, comparing first-episode psychosis to chronic patients, demonstrated that disease duration also influenced the extent of cytokine alterations. Risperidone treatment significantly lowered levels of IL-6 and TNF-α in patients with chronic disease, while this effect was not evident in individuals experiencing their first episode of psychosis [133]. Future investigations should encompass a broad range of medications, along with multiple follow-up assessments for a longer duration*”.

**References**:

1.	Brown, D., *A Review of the PubMed PICO Tool: Using Evidence-Based Practice in Health Education.* Health Promot Pract, 2020. **21**(4): p. 496-498.

2.	Openshaw, R.L., et al., *JNK signalling mediates aspects of maternal immune activation: importance of maternal genotype in relation to schizophrenia risk.* JOURNAL OF NEUROINFLAMMATION, 2019. **16**.

3.	Oschwald, A., et al., *CNS Macrophages and Infant Infections.* Frontiers in Immunology, 2020. **11**.

4.	Paudel, Y.N., et al., *Role of inflammation in epilepsy and neurobehavioral comorbidities: Implication for therapy.* European Journal of Pharmacology, 2018. **837**: p. 145-155.

5.	Pusceddu, M.M. and J.M. Del Bas, *The role of the gut microbiota in the pathophysiology of mental and neurological disorders.* Psychiatric Genetics, 2020. **30**(4): p. 87-100.

6.	Ribeiro, B.M.M., et al., *N-3 polyunsaturated fatty acids and clozapine abrogates poly I: C-induced immune alterations in primary hippocampal neurons.* Progress in Neuro-Psychopharmacology and Biological Psychiatry, 2019. **90**: p. 186-196.

7.	Sancho-Balsells, A., et al., *Lack of Helios During Neural Development Induces Adult Schizophrenia-Like Behaviors Associated With Aberrant Levels of the TRIF-Recruiter Protein WDFY1.* Frontiers in Cellular Neuroscience, 2020. **14**.

8.	Slusarczyk, J., et al., *Targeting the NLRP3 Inflammasome-Related Pathways via Tianeptine Treatment-Suppressed Microglia Polarization to the M1 Phenotype in Lipopolysaccharide-Stimulated Cultures.* INTERNATIONAL JOURNAL OF MOLECULAR SCIENCES, 2018. **19**(7).

9.	Tian, L. and M.E. Tremblay, *Importance of the immune system in mediating plasticity of the brain and behavior.* Progress in Neuro-Psychopharmacology and Biological Psychiatry, 2017. **79**: p. 1-2.

10.	van Rees, G.F., et al., *Evidence of microglial activation following exposure to serum from first-onset drug-naïve schizophrenia patients.* Brain, Behavior, and Immunity, 2018. **67**: p. 364-373.

11.	Lopatina, O.L., et al., *CD157 and Brain Immune System in (Patho)physiological Conditions: Focus on Brain Plasticity.* Frontiers in Immunology, 2020. **11**.

12.	López-González, I., et al., *Neuroinflammation in the dorsolateral prefrontal cortex in elderly chronic schizophrenia.* Eur Neuropsychopharmacol, 2019. **29**(3): p. 384-396.

13.	Lu-Culligan, A. and A. Iwasaki, *The Role of Immune Factors in Shaping Fetal Neurodevelopment*. 2020. p. 441-468.

14.	MacDowell, K.S., et al., *Differential regulation of the TLR4 signalling pathway in post-mortem prefrontal cortex and cerebellum in chronic schizophrenia: Relationship with SP transcription factors.* Prog Neuropsychopharmacol Biol Psychiatry, 2017. **79**: p. 481-492.

15.	Mantere, O., et al., *Immunomodulatory effects of antipsychotic treatment on gene expression in first-episode psychosis.* Journal of Psychiatric Research, 2019. **109**: p. 18-26.

16.	Morris, G., et al., *Emerging role of innate B1 cells in the pathophysiology of autoimmune and neuroimmune diseases: Association with inflammation, oxidative and nitrosative stress and autoimmune responses.* Pharmacol Res, 2019. **148**: p. 104408.

17.	Pardo-de-Santayana, G., et al., *Active psychosis and pro-inflammatory cytokines in first-episode of psychosis.* J Psychiatr Res, 2021. **134**: p. 150-157.

18.	Leon-Rodriguez, A., et al., *Anxiety-like behavior and microglial activation in the amygdala after acute neuroinflammation induced by microbial neuraminidase.* SCIENTIFIC REPORTS, 2022. **12**(1).

19.	Aflouk, Y., et al., *Association between TLR2 polymorphisms (- 196-174 Ins/Del, R677W, R753Q, and P631H) and schizophrenia in a Tunisian population.* Immunol Res, 2021. **69**(6): p. 541-552.

20.	Mou, T.C.M., et al., *Association of complement component 4 with neuroimmune abnormalities in the subventricular zone in schizophrenia and autism spectrum disorders.* Neurobiology of Disease, 2022. **173**.

21.	Malashenkova, I.K., et al., *The association of inflammatory status and immunological parameters with single-nucleotide polymorphisms of cytokine and Toll-like receptor genes in patients with schizophrenia.* Research Results in Biomedicine, 2022. **8**(2): p. 148-163.

22.	Gurung, J., et al., *Association of TLR-4 896A/G, TLR-4 1196C/T, and TLR-9 C/T polymorphism with schizophrenia in Indian Bengalee patient.* Indian J Psychiatry, 2022. **64**(6): p. 579-587.

23.	Sharma, I., et al., *Association of toll-like receptor 2 gene polymorphism (rs3804099) with susceptibility to Schizophrenia risk in the Dogra population of Jammu region, North India.* European Journal of Psychiatry, 2022. **36**(2): p. 106-113.

24.	Maly, I.V., M.J. Morales, and M.V. Pletnikov, *Astrocyte Bioenergetics and Major Psychiatric Disorders*. 2021. p. 173-227.

25.	Fern, et al., *Charting the proteome landscape in major psychiatric disorders: From biomarkers to biological pathways towards drug discovery.* Eur Neuropsychopharmacol, 2022. **61**: p. 43-59.

26.	Wang, Y., et al., *Cognitive impairment in psychiatric diseases: Biomarkers of diagnosis, treatment, and prevention.* Frontiers in Cellular Neuroscience, 2022. **16**.

27.	Elgueta, D., et al., *Consequences of Viral Infection and Cytokine Production During Pregnancy on Brain Development in Offspring.* Frontiers in Immunology, 2022. **13**.

28.	Kwon, J., et al., *Differential Effects of Toll-Like Receptor Activation and Differential Mediation by MAP Kinases of Immune Responses in Microglial Cells.* CELLULAR AND MOLECULAR NEUROBIOLOGY, 2022. **42**(8): p. 2655-2671.

29.	Li, N., et al., *Dysfunctional monocytic toll-like receptor 4 signaling pathway and cognitive deficits in chronic schizophrenia patients with tardive dyskinesia.* NEUROSCIENCE LETTERS, 2022. **777**.

30.	King, S., et al., *Early life Adversity, functional connectivity and cognitive performance in Schizophrenia: The mediating role of IL-6.* Brain Behav Immun, 2021. **98**: p. 388-396.

31.	Xu, H., et al., *Editorial: Cognitive Dysfunctions in Psychiatric Disorders: Brain-Immune Interaction Mechanisms and Integrative Therapeutic Approaches.* Frontiers in Integrative Neuroscience, 2021. **15**.

32.	Arslan, A., O. Aydin, and F.P. Çökmüş, *Editorial: The inflammation markers in schizophrenia and bipolar disorder: Do we have promising results?* Frontiers in Psychiatry, 2023. **13**.

33.	Robinson, N. and S.E. Bergen, *Environmental Risk Factors for Schizophrenia and Bipolar Disorder and Their Relationship to Genetic Risk: Current Knowledge and Future Directions.* Frontiers in Genetics, 2021. **12**.

34.	Maes, M., et al., *First Episode Psychosis and Schizophrenia Are Systemic Neuro-Immune Disorders Triggered by a Biotic Stimulus in Individuals with Reduced Immune Regulation and Neuroprotection.* Cells, 2021. **10**(11).

35.	Misiak, B., et al., *Gut microbiota in psychiatric disorders: Better understanding or more complexity to be resolved?* Progress in Neuro-Psychopharmacology and Biological Psychiatry, 2021. **110**.

36.	Wang, X., et al., *Human endogenous retrovirus W family envelope protein (HERV-W env) facilitates the production of TNF-α and IL-10 by inhibiting MyD88s in glial cells.* Arch Virol, 2021. **166**(4): p. 1035-1045.

37.	Xie, M., et al., *Identifying crucial biomarkers in peripheral blood of schizophrenia and screening therapeutic agents by comprehensive bioinformatics analysis.* Journal of Psychiatric Research, 2022. **152**: p. 86-96.

38.	Ermakov, E.A., et al., *Immune System Abnormalities in Schizophrenia: An Integrative View and Translational Perspectives.* Frontiers in Psychiatry, 2022. **13**.

39.	Rhoades, R., et al., *Impact of SARS-CoV-2 on Host Factors Involved in Mental Disorders.* Frontiers in Microbiology, 2022. **13**.

40.	Oseghale, O., et al., *Influenza Virus Infection during Pregnancy as a Trigger of Acute and Chronic Complications.* Viruses, 2022. **14**(12).

41.	Chen, J., et al., *Involvement of TCF7L2 in generation of morphine-induced antinociceptive tolerance and hyperalgesia by modulating TLR4/ NF-κB/NLRP3 in microglia.* Toxicology and Applied Pharmacology, 2021. **416**.

42.	Snijders, G.J.L.J., et al., *A loss of mature microglial markers without immune activation in schizophrenia.* GLIA, 2021. **69**(5): p. 1251-1267.

43.	Kwon, H.K., G.B. Choi, and J.R. Huh, *Maternal inflammation and its ramifications on fetal neurodevelopment.* Trends in Immunology, 2022. **43**(3): p. 230-244.

44.	Ibrahim, I., et al., *Modulatory Effect of Gut Microbiota on the Gut-Brain, Gut-Bone Axes, and the Impact of Cannabinoids.* Metabolites, 2022. **12**(12).

45.	Supino, D., et al., *Negative Regulation of the IL-1 System by IL-1R2 and IL-1R8: Relevance in Pathophysiology and Disease.* Frontiers in Immunology, 2022. **13**.

46.	Morozova, A., et al., *Neurobiological Highlights of Cognitive Impairment in Psychiatric Disorders.* International Journal of Molecular Sciences, 2022. **23**(3).

47.	Murphy, C.E., et al., *Peripheral NF-κB dysregulation in people with schizophrenia drives inflammation: putative anti-inflammatory functions of NF-κB kinases.* Translational Psychiatry, 2022. **12**(1).

48.	Zhang, Y., et al., *A Possible Causal Involvement of Neuroinflammatory, Purinergic P2X7 Receptors in Psychiatric Disorders.* Curr Neuropharmacol, 2022. **20**(11): p. 2142-2155.

49.	Andrade, C.A., A.M. Kalergis, and K. Bohmwald, *Potential Neurocognitive Symptoms Due to Respiratory Syncytial Virus Infection.* Pathogens, 2022. **11**(1).

50.	Ozaki, A., Y. Yamawaki, and G. Ohtsuki, *Psychosis symptoms following aberrant immunity in the brain.* Neural Regeneration Research, 2021. **16**(3): p. 512-513.

51.	Li, H., et al., *The relationship between TLR4/NF-κB/IL-1β signaling, cognitive impairment, and white-matter integrity in patients with stable chronic schizophrenia.* Frontiers in Psychiatry, 2022. **13**.

52.	Kelly, J.R., et al., *The role of the gut microbiome in the development of schizophrenia.* Schizophrenia Research, 2021. **234**: p. 4-23.

53.	Carnac, T., *Schizophrenia Hypothesis: Autonomic Nervous System Dysregulation of Fetal and Adult Immune Tolerance.* Front Syst Neurosci, 2022. **16**: p. 844383.

54.	Langeh, U. and S. Singh, *Targeting s100b protein as a surrogate biomarker and its role in various neurological disorders.* Current Neuropharmacology, 2021. **19**(2): p. 265-277.

55.	Ketharanathan, T., et al., *TOLL-LIKE RECEPTOR (TLR) PATHWAY EXPRESSION IN DORSOLATERAL PREFRONTAL (BA46) AND ORBITOFRONTAL (BA11) CORTICES IN SCHIZOPHRENIA AND MOOD DISORDER.* AUSTRALIAN AND NEW ZEALAND JOURNAL OF PSYCHIATRY, 2022. **56**(1): p. 222-222.

56.	Mostafa, M., et al., *Toll-Like Receptor 4 Gene Polymorphisms and Susceptibility to Schizophrenia: A Case-Control Study.* Immunol Invest, 2022. **51**(7): p. 2009-2024.

57.	Massarali, A., et al., *Virus-Induced Maternal Immune Activation as an Environmental Factor in the Etiology of Autism and Schizophrenia.* Frontiers in Neuroscience, 2022. **16**.

58.	Kéri, S., C. Szabó, and O. Kelemen, *Antipsychotics influence Toll-like receptor (TLR) expression and its relationship with cognitive functions in schizophrenia.* Brain Behav Immun, 2017. **62**: p. 256-264.

59.	Kéri, S., C. Szabó, and O. Kelemen, *Uniting the neurodevelopmental and immunological hypotheses: Neuregulin 1 receptor ErbB and Toll-like receptor activation in first-episode schizophrenia.* Sci Rep, 2017. **7**(1): p. 4147.

60.	Kozłowska, E., et al., *The expression of toll-like receptors in peripheral blood mononuclear cells is altered in schizophrenia.* Psychiatry Res, 2019. **272**: p. 540-550.

61.	Li, C.J., *Oxidative stress and mitochondrial dysfunction in human diseases: Pathophysiology, predictive biomarkers, therapeutic.* Biomolecules, 2020. **10**(11): p. 1-3.

62.	Li, H. and S. Kéri, *TLR4 (Toll-like receptor-4) expression and frontal-cingulate volumes in schizophrenia.* Ideggyogy Sz, 2020. **73**(9): p. 303-308.

63.	Li, Z., et al., *The TMEM106B FTLD-protective variant, rs1990621, is also associated with increased neuronal proportion.* Acta Neuropathologica, 2020. **139**(1): p. 45-61.

64.	Liao, Y., et al., *Maternal Murine Cytomegalovirus Infection during Pregnancy Up-regulates the Gene Expression of Toll-like Receptor 2 and 4 in Placenta.* CURRENT MEDICAL SCIENCE, 2018. **38**(4): p. 632-639.

65.	Wadhawan, A., et al., *Periodontal pathogens and neuropsychiatric health.* Current Topics in Medicinal Chemistry, 2020. **20**(15): p. 1353-1397.

66.	Wang, X., J. Huang, and F. Zhu, *Human endogenous retroviral envelope protein Syncytin-1 and inflammatory abnormalities in neuropsychological diseases.* Frontiers in Psychiatry, 2018. **9**.

67.	Wang, X., et al., *Syncytin-1, an endogenous retroviral protein, triggers the activation of CRP via TLR3 signal cascade in glial cells.* Brain, Behavior, and Immunity, 2018. **67**: p. 324-334.

68.	Chaumette, B., et al., *Longitudinal Analyses of Blood Transcriptome During Conversion to Psychosis.* Schizophr Bull, 2019. **45**(1): p. 247-255.

69.	Chen, S., et al., *More dampened monocytic Toll-like receptor 4 response to lipopolysaccharide and its association with cognitive function in Chinese Han first-episode patients with schizophrenia.* Schizophr Res, 2019. **206**: p. 300-306.

70.	Ermakov, E.A., et al., *Natural Catalytic IgGs Hydrolyzing Histones in Schizophrenia: Are They the Link between Humoral Immunity and Inflammation?* Int J Mol Sci, 2020. **21**(19).

71.	Gruchot, J., D. Kremer, and P. Küry, *Neural cell responses upon exposure to human endogenous retroviruses.* Frontiers in Genetics, 2019. **10**.

72.	Herron, J.W., L. Nerurkar, and J. Cavanagh, *Neuroimmune biomarkers in mental illness*. 2018. p. 45-78.

73.	Huang, Q., et al., *Microbiota-immune system interactions in human neurological disorders.* CNS and Neurological Disorders - Drug Targets, 2020. **19**(7): p. 509-526.

74.	Juncal-Ruiz, M., et al., *Expression and Functionality Study of 9 Toll-Like Receptors in 33 Drug-Naive Non-Affective First Episode Psychosis Individuals: A 3-Month Study.* INTERNATIONAL JOURNAL OF MOLECULAR SCIENCES, 2020. **21**(17).

75.	Kelly, J.R., et al., *Cross talk: The microbiota and neurodevelopmental disorders.* Frontiers in Neuroscience, 2017. **11**.

76.	Kéri, S., C. Szabó, and O. Kelemen, *Erratum: Uniting the neurodevelopmental and immunological hypotheses: Neuregulin 1 receptor ErbB and Toll-like receptor activation in first-episode schizophrenia.* Sci Rep, 2017. **7**(1): p. 9758.

77.	Keri, S., C. Szabo, and O. Kelemen, *Uniting the neurodevelopmental and immunological hypotheses: Neuregulin 1 receptor ErbB and Toll-like receptor activation in first-episode schizophrenia (vol 7, 4141, 2017).* SCIENTIFIC REPORTS, 2017. **7**.

78.	Ashdown, H., et al., *The role of cytokines in mediating effects of prenatal infection on the fetus: implications for schizophrenia.* MOLECULAR PSYCHIATRY, 2006. **11**(1): p. 47-55.

79.	Ibi, D., et al., *Neonatal polyI:C treatment in mice results in schizophrenia-like behavioral and neurochemical abnormalities in adulthood.* NEUROSCIENCE RESEARCH, 2009. **64**(3): p. 297-305.

80.	Chang, S.H., et al., *Expression of anti-cardiolipin antibodies and inflammatory associated factors in patients with schizophrenia.* Psychiatry Res, 2011. **187**(3): p. 341-6.

81.	McKernan, D.P., et al., *ALTERED TOLL-LIKE RECEPTOR RESPONSES IN SCHIZOPHRENIA AND BIPOLAR DISORDER PATIENTS.* IRISH JOURNAL OF MEDICAL SCIENCE, 2011. **180**: p. 63-63.

82.	McKernan, D.P., et al., *Enhanced peripheral toll-like receptor responses in psychosis: further evidence of a pro-inflammatory phenotype.* Transl Psychiatry, 2011. **1**(8): p. e36.

83.	Bolon, B., *Cellular and Molecular Mechanisms of Autoimmune Disease.* Toxicologic Pathology, 2012. **40**(2): p. 216-229.

84.	Krause, D., et al., *Monocytic HLA DR antigens in schizophrenic patients.* Neurosci Res, 2012. **72**(1): p. 87-93.

85.	Lee, S.M., et al., *Toll-like receptor 2 polymorphisms are associated with poor concentration in schizophrenia patients in a Korean population.* EUROPEAN NEUROPSYCHOPHARMACOLOGY, 2012. **22**: p. S156-S157.

86.	Müller, N., et al., *Impaired monocyte activation in schizophrenia.* Psychiatry Res, 2012. **198**(3): p. 341-6.

87.	Aguirre, A., et al., *Possible involvement of TLRs and hemichannels in stress-induced CNS dysfunction via mastocytes, and glia activation.* Mediators Inflamm, 2013. **2013**: p. 893521.

88.	Barichello, T., et al., *Pathophysiology of bacterial infection of the central nervous system and its putative role in the pathogenesis of behavioral changes.* Revista Brasileira de Psiquiatria, 2013. **35**(1): p. 81-87.

89.	Chew, L.J., P. Fusar-Poli, and T. Schmitz, *Oligodendroglial Alterations and the Role of Microglia in White Matter Injury: Relevance to Schizophrenia.* DEVELOPMENTAL NEUROSCIENCE, 2013. **35**(2): p. 102-129.

90.	Hopper, A.T., et al., *Neuroinflammation in mood disorders: Mechanisms and drug targets*. 2013. p. 317-331.

91.	Kang, W.S., et al., *Association between genetic polymorphisms of Toll-like receptor 2 (TLR2) and schizophrenia in the Korean population.* Gene, 2013. **526**(2): p. 182-6.

92.	Liu, H.Y., et al., *TLR7 Negatively Regulates Dendrite Outgrowth through the Myd88-c-Fos-IL-6 Pathway.* JOURNAL OF NEUROSCIENCE, 2013. **33**(28): p. 11479-11493.

93.	Lucas, K. and M. Maes, *Role of the Toll Like receptor (TLR) radical cycle in chronic inflammation: possible treatments targeting the TLR4 pathway.* Mol Neurobiol, 2013. **48**(1): p. 190-204.

94.	Shastri, A., D.M. Bonifati, and U. Kishore, *Innate immunity and neuroinflammation.* Mediators of Inflammation, 2013. **2013**.

95.	Dammann, O. and A. Leviton, *Intermittent or sustained systemic inflammation and the preterm brain.* Pediatric Research, 2014. **75**(3): p. 376-380.

96.	Lin, C.W., et al., *Sarm1 deficiency impairs synaptic function and leads to behavioral deficits, which can be ameliorated by an mGluR allosteric modulator.* Front Cell Neurosci, 2014. **8**: p. 87.

97.	Nagai, T., *Effects of Genetic and Environmental Factors on Neuropsychological Development.* YAKUGAKU ZASSHI-JOURNAL OF THE PHARMACEUTICAL SOCIETY OF JAPAN, 2014. **134**(10): p. 1029-1035.

98.	Xia, Y.C., et al., *Influenza vaccination during early pregnancy contributes to neurogenesis and behavioral function in offspring.* BRAIN BEHAVIOR AND IMMUNITY, 2014. **42**: p. 212-221.

99.	Yu, H., et al., *Protein-interaction-network-based analysis for genome-wide association analysis of schizophrenia in Han Chinese population.* JOURNAL OF PSYCHIATRIC RESEARCH, 2014. **50**: p. 73-78.

100.	Crisafulli, C., et al., *A molecular pathway analysis informs the genetic background at risk for schizophrenia.* Prog Neuropsychopharmacol Biol Psychiatry, 2015. **59**: p. 21-30.

101.	de Baumont, A., et al., *Innate immune response is differentially dysregulated between bipolar disease and schizophrenia.* Schizophrenia Research, 2015. **161**(2): p. 215-221.

102.	Szabo, A., *Psychedelics and Immunomodulation: Novel Approaches and Therapeutic Opportunities.* Front Immunol, 2015. **6**: p. 358.

103.	Bernstein, H.G., Y. Piontkewitz, and G. Keilhoff, *Commentary: Maternal immune activation evoked by polyinosinic: Polycytidylic acid does not evoke microglial cell activation in the embryo.* Frontiers in Cellular Neuroscience, 2016. **10**.

104.	Caso, J.R., et al., *The Microbiota and Gut-Brain Axis: Contributions to the Immunopathogenesis of Schizophrenia.* Curr Pharm Des, 2016. **22**(40): p. 6122-6133.

105.	Chrobak, A.A., J. Nowakowski, and D. Dudek, *Interactions between the gut microbiome and the central nervous system and their role in schizophrenia, bipolar disorder and depression.* Archives of Psychiatry and Psychotherapy, 2016. **18**(2): p. 5-11.

106.	García Bueno, B., et al., *Innate immune receptor Toll-like receptor 4 signalling in neuropsychiatric diseases.* Neuroscience and Biobehavioral Reviews, 2016. **64**: p. 134-147.

107.	García-Bueno, B., et al., *Evidence of activation of the Toll-like receptor-4 proinflammatory pathway in patients with schizophrenia.* J Psychiatry Neurosci, 2016. **41**(3): p. E46-55.

108.	Lee, Y., et al., *Activation of maternal toll-like receptor-2 causes social deficits and memory impairment in female offspring.* NEUROREPORT, 2016. **27**(4): p. 224-229.

109.	Orhan, F., et al., *Tryptophan Metabolism Along the Kynurenine Pathway Downstream of Toll-like Receptor Stimulation in Peripheral Monocytes.* SCANDINAVIAN JOURNAL OF IMMUNOLOGY, 2016. **84**(5): p. 262-271.

110.	Severance, E.G., R.H. Yolken, and W.W. Eaton, *Autoimmune diseases, gastrointestinal disorders and the microbiome in schizophrenia: more than a gut feeling.* SCHIZOPHRENIA RESEARCH, 2016. **176**(1): p. 23-35.

111.	Sharma, A. and R. Goyal, *Experimental brain ischemic preconditioning: A concept to putative targets.* CNS and Neurological Disorders - Drug Targets, 2016. **15**(4): p. 489-495.

112.	Adorjan, I., et al., *Evidence for Decreased Density of Calretinin-Immunopositive Neurons in the Caudate Nucleus in Patients With Schizophrenia.* Frontiers in Neuroanatomy, 2020. **14**.

113.	Alam, R., H.M. Abdolmaleky, and J.R. Zhou, *Microbiome, inflammation, epigenetic alterations, and mental diseases.* American Journal of Medical Genetics, Part B: Neuropsychiatric Genetics, 2017. **174**(6): p. 651-660.

114.	Ardalan, M., et al., *Sex-dependent effects of perinatal inflammation on the brain: Implication for neuro-psychiatric disorders.* International Journal of Molecular Sciences, 2019. **20**(9).

115.	Balaji, R., et al., *Pattern of expression of Toll like receptor (TLR)-3 and -4 genes in drug-naïve and antipsychotic treated patients diagnosed with schizophrenia.* Psychiatry Res, 2020. **285**: p. 112727.

116.	Chase, K.A., et al., *Traumagenics: At the intersect of childhood trauma, immunity and psychosis.* Psychiatry Research, 2019. **273**: p. 369-377.

117.	Zagorska, A., et al., *From probiotics to psychobiotics - the gut-brain axis in psychiatric disorders.* BENEFICIAL MICROBES, 2020. **11**(8): p. 717-732.

118.	Lashgari, N.A., et al., *TLR/mTOR inflammatory signaling pathway: novel insight for the treatment of schizophrenia.* Can J Physiol Pharmacol, 2024. **102**(3): p. 150-160.

119.	Chaudhary, A., et al., *The Emerging Role of Toll-Like Receptor-Mediated Neuroinflammatory Signals in Psychiatric Disorders and Acquired Epilepsy.* Mol Neurobiol, 2024. **61**(3): p. 1527-1542.

120.	Ayari, F., et al., *Association between genetic variants of TLR2, TLR4, TLR9 and schizophrenia.* Encephale, 2024. **50**(2): p. 178-184.

121.	Tseng, C.C., et al., *Aberrant Histone Modification of TNFAIP3, TLR4, TNIP2, miR-146a, and miR-155 in Major Depressive Disorder.* Mol Neurobiol, 2023. **60**(8): p. 4753-4760.

122.	Sotelo-Ramírez, C.E., et al., *Toll-Like Receptor (TLR) 1, 2, and 6 Gene Polymorphisms Support Evidence of Innate Immune Factors in Schizophrenia.* Neuropsychiatr Dis Treat, 2023. **19**: p. 2353-2361.

123.	Tsai, S.Y., et al., *Increased proportions of circulating pro-inflammatory monocytes and macrophages expressing toll-like receptor 4 in individuals with schizophrenia and bipolar disorder receiving medication.* Psychiatry Clin Neurosci, 2023. **77**(12): p. 672-673.

124.	Patlola, S.R., G. Donohoe, and D.P. McKernan, *Counting the Toll of Inflammation on Schizophrenia-A Potential Role for Toll-like Receptors.* Biomolecules, 2023. **13**(8).

125.	Chung, I.H., et al., *Whole Genome Sequencing Revealed Inherited Rare Oligogenic Variants Contributing to Schizophrenia and Major Depressive Disorder in Two Families.* Int J Mol Sci, 2023. **24**(14).

126.	Murphy, C.E., A.K. Walker, and C.S. Weickert, *Neuroinflammation in schizophrenia: the role of nuclear factor kappa B.* Translational Psychiatry, 2021. **11**(1): p. 528.

127.	Xia, P., et al., *Research progress on Toll-like receptor signal transduction and its roles in antimicrobial immune responses.* Applied Microbiology and Biotechnology, 2021. **105**(13): p. 5341-5355.

128.	Nielsen, P.R., M.E. Benros, and P.B. Mortensen, *Hospital Contacts With Infection and Risk of Schizophrenia: A Population-Based Cohort Study With Linkage of Danish National Registers.* Schizophrenia Bulletin, 2014. **40**(6): p. 1526-1532.

129.	Weickert, T.W., et al., *Toll-Like Receptor mRNA Levels in Schizophrenia: Association With Complement Factors and Cingulate Gyrus Cortical Thinning.* Schizophrenia Bulletin, 2023: p. sbad171.

130.	Kéri, S., C. Szabó, and O. Kelemen, *Antipsychotics influence Toll-like receptor (TLR) expression and its relationship with cognitive functions in schizophrenia.* Brain Behav Immun, 2016. **62**: p. 256-264.

131.	Li, H., et al., *The relationship between TLR4/NF-κB/IL-1β signaling, cognitive impairment, and white-matter integrity in patients with stable chronic schizophrenia.* Front Psychiatry, 2022. **13**: p. 966657.

132.	Meyer, U., M.J. Schwarz, and N. Müller, *Inflammatory processes in schizophrenia: a promising neuroimmunological target for the treatment of negative/cognitive symptoms and beyond.* Pharmacol Ther, 2011. **132**(1): p. 96-110.

133.	Patlola, S.R., G. Donohoe, and D.P. McKernan, *Anti-inflammatory effects of 2nd generation antipsychotics in patients with schizophrenia: A systematic review and meta-analysis.* Journal of Psychiatric Research, 2023. **160**: p. 126-136.

1. Exclusion reason(s) is reported for the excluded studies. Name of data extractors and date of data extraction are reported for the included studies.
2. Exclusion reason(s) is reported for the excluded studies. Name of data extractors and date of data extraction are reported for the included studies.
